# Prognostic factors for mental wellbeing in prostate cancer: A systematic review and meta‐analysis

**DOI:** 10.1002/pon.6225

**Published:** 2023-10-03

**Authors:** Neel Vyas, Oliver Brunckhorst, Jack B. Fanshawe, Robert Stewart, Prokar Dasgupta, Kamran Ahmed

**Affiliations:** ^1^ MRC Centre for Transplantation Guy's Hospital Campus King's College London King's Health Partners London UK; ^2^ Urology Centre Guy's and St. Thomas' NHS Foundation Trust King's Health Partners London London UK; ^3^ King's College London Institute of Psychiatry, Psychology and Neuroscience London UK; ^4^ South London and Maudsley NHS Foundation Trust London UK; ^5^ Department of Urology Sheikh Khalifa Medical City Abu Dhabi UAE; ^6^ Khalifa University Abu Dhabi UAE

**Keywords:** anxiety, body image, cancer, depression, fear of cancer recurrence, masculinity, mental wellbeing, oncology, prostate cancer, quality of life

## Abstract

**Objectives:**

To evaluate the evidence base for patient, oncological, and treatment prognostic factors associated with multiple mental wellbeing outcomes in prostate cancer patients.

**Methods:**

We performed a literature search of MEDLINE, EMBASE, and CINAHL databases including studies evaluating patient, oncological, or treatment factors against one of five mental wellbeing outcomes; depression, anxiety, fear of cancer recurrence, masculinity, and body image perception. Data synthesis included a random effects meta‐analysis for the prognostic effect of individual factors if sufficient homogenous data was available, with a structured narrative synthesis where this was not possible.

**Results:**

A final 62 articles were included. Older age was associated with a reducing odds of depression (OR 0.97, *p* = 0.04), with little evidence of effect for other outcomes. Additionally, baseline mental health status was related to depression and increasing time since diagnosis was associated with reducing fear of recurrence, albeith with low certainty of evidence. However, few other patient or oncological factors demonstrated any coherent relationship with any wellbeing outcome. Androgen deprivation therapy was associated with increased depression (HR 1.65, 95% CI 1.41–1.92, *p* < 0.01) and anxiety, however, little difference was seen between other treatment options. Overall, whilst numerous factors were identified, most were evaluated by single studies with few evaluating masculinity and body image outcomes.

**Conclusion:**

We highlight the existing evidence for prognostic factors in mental wellbeing outcomes in prostate cancer, allowing us to consider high‐risk groups of patients for preventative and treatment measures. However, the current evidence is heterogenous with further work required exploring less conclusive factors and outcomes.

## 1 BACKGROUND

Prostate Cancer (PC) is the second most common malignancy in men worldwide with over 1.2 million yearly cases globally.^1^ With life expectancy and detection rates increasing, this figure is also expected to rise.^2^ When this is combined with the overall high and increasing survival rates of over 80% at 5 years, there are now increasing number of individuals are now living with and beyond their disease.^3^, ^4^ This necessitates a drive to improve survivorship care for these patients, to ensure they are not only living longer, but also maintain a good quality of life after treatment.^5^, ^6^

Much of this effort remains focussed on the physical sequelae of disease, which dominates the existing literature. However, the psychosocial consequences of disease are becoming increasingly apparent. Mental health conditions are a common issue, with an estimated 17% of patients experiencing significant depressive or anxiety symptoms after diagnosis.^7^ But mental wellbeing in PC consists of more than clinical mental health disorders, with several other important distinct constructs.^8^ Fear of cancer recurrence (FCR) is one of these, commonly defined as a “Fear, worry, or concern relating to the possibility that cancer will come back or progress”.^9^ FCR is often cited as the most unmet cancer need during survivorship affecting a high proportion of patients.^10^, ^11^ Other important outcomes include body image and masculinity perception. Body image disturbance includes a displeasure or distress with a perceived or actual change in body appearance or function.^12^ Masculinity is broadly a social concept of gender which is strongly influenced by historical, social and cultural factors.^13^ Many definitions exists with several incorporating traditional views centred around hegemonic masculine ideals defined by a set of idealised practices including restricted emotional expression, power and success, stoicism, and heterosexism, with many existing measurement tools centred around these.^14^, ^15^, ^16^ However, increasingly varying definitions are being utilised with evolving societal ideas around what masculinity is, with the inclusive masculine theory an example.^17^ Both body image and masculinity issues have been highlighted as being of particular importance to patients with PC due to the frequent sexual and urinary complications of treatment.^18^

However, in response to evolving ideals of masculinity, particularly surrounding reducing ‘homohysteria’ (i.e. the fear of being homosexualised) and decreasing patriarchal views, the inclusive masculine theory was derived and refined by Anderson.^17^ This follows that men now are less afraid to develop softer, more expressive relationships with more inclusive ideals of what it means to be a man. This means less rigid and predefined concepts are included within what it means ‘to be a man’ for different individuals. For this thesis various theories surrounding masculinity were considered due to the varying spectrum of what masculinity is to differing individuals. Whilst ultimately it was considered as anything which affected their perception of their own gender, this was based predominantly around the inclusive masculine theory to reflect more modern and inclusive societal views, whilst acknowledging elements from traditional hegemonic masculinities.

Whilst we know the overall impact of PC on individuals mental wellbeing, less is known about individual prognostic factors for these outcomes. Some factors such as Androgen Deprivation Therapy (ADT) appear to be associated with mental health outcomes such as depression; however, there is little consensus on other patient, oncological or treatment factors.^19^ This is particularly true for non‐mental health outcomes such as FCR, body image and masculinity, with no previous dedicated review of prognostic factors evaluating these. A clearer understanding of factors associated with mental wellbeing is imperative, allowing us to improve patient support for those at‐risk, and highlight areas for future research, including the design and evaluation of pre‐ and post‐treatment interventions to mitigate these factors.^20^ Therefore, this review aimed to evaluate the evidence for individual patient, oncological and treatment prognostic factors associated with mental wellbeing outcomes in patients with PC, including depression, anxiety, FCR, masculinity, and body image disturbance.

## 2 METHODS

The reporting of this review was conducted in line with the ‘Preferred Reporting Items for Systematic reviews and Meta‐Analyses’ (PRISMA) and PRISMA‐literature search extension (PRISMA‐S) guideline.^21^, ^22^ Where a meta‐analysis was not possible the Synthesis Without Meta‐analysis (SWiM) reporting guidelines were used.^23^ The conduct of this review was set within the PROGRESS (PROGnosis RESearch Strategy) framework with *a* prior protocol registered on the PROSPERO database (CRD42021297396).^20^, ^24^

### 2.1 Study eligibility criteria

Inclusion criteria were any studies evaluating a PC population, with an index demographic patient, treatment modality, or oncological characteristics prognostic factor against a relevant comparator defined as an immediately comparable factor within the same category (e.g., comparison of differing ethnicities, stage at diagnosis, or treatment undergone for patient, oncological and treatment factors respectively) for any one of our defined mental wellbeing outcomes. These included depression, anxiety, FCR, masculinity and body image perception outcomes. The definitions of some of these have been described within the background section, however, for masculinity in view of these differing definitions utilised within the literature no pre‐set inclusion requirement for a specific definition of this was set. These specific outcomes were selected following numerous previous research studies from the authors conducted as background work. Previously, it has been highlighted that mental wellbeing is poorly defined within the literature.^8^ Therefore, multiple separate reviews and qualitative patient interviews were conducted, with a triangulation approach of these in combination used to better define this, selecting outcomes seen to be prevalent, severe and of importance to patients leading to five outcomes being selected as set out in this review.^7^, ^11^, ^18^, ^25^, ^26^

No restrictions were set on post‐diagnosis timing with any clinical setting included. Studies had to utilise either a previously validated psychometric tool to assess the outcome of interest, or diagnostic coding data following clinical diagnosis for depression and anxiety outcomes. We included both observational and interventional studies of either prospective and retrospective design as long as they were longitudinal in nature, with no restrictions on minimum follow up duration for the studies.

Studies were excluded if they were non‐English with no translation available, conference abstracts, comment articles or reviews. Cross‐sectional studies were excluded to improve data quality with these studies being less appropriate for the evaluation of prognostic factors for the development of an outcome. Where a study included a mixed cancer population, this was excluded if individual results for the PC population were not available. Additionally, studies which did not exclusively evaluate previously listed outcomes were excluded, including those assessing composite outcomes (mixed anxiety‐depression) or emotional/cancer specific distress, or broader mental/emotional health due to varying definitions and overlap seen for these.

### 2.2 Information sources and search strategy

A comprehensive literature search was carried out from inception to 17/08/2022 using MEDLINE, EMBASE and CINAHL databases. Grey literature was searched for ongoing studies via the ClinicalTrials.gov database, with authors contacted for any preliminary data available. Additionally, a reference review of included studies was conducted for any further pertinent articles. A piloted search strategy was utilised which included a mixture of key words, MeSH terms, and related synonyms for PC, mental wellbeing outcomes of interest, and prognostic factors (Supplementary Material S1). In view of the presumed limited availability of longitudinal studies for many outcomes and the broad study aims to evaluate multiple factors, the search strategy and terms was maintained broad to ensure as wide a scope as possible within the search results.

### 2.3 Study selection

Following removal of duplication two independent reviewers (NV and OB) screened search results against study eligibility criteria via title, abstract and full text evaluation using Rayyan software to help with reference management.^27^ Discrepancies between reviewers were discussed until full agreement was reached.

### 2.4 Data collection and data items

Data extraction was conducted by two reviewers (NV and OB) independently utilising a pre‐defined and piloted extraction sheet based on the CHARMS‐PF checklist.^24^ Study characteristics extracted from each article included: author, country, study dates, study design, population, mean/median age of patients, follow up period, treatment received and stages of disease. Additionally, analysis methods for the outcome of interest were extracted, including diagnostic criteria utilised and statistical method of analysis for evaluation of the prognostic factor. This included if a univariate or multivariate analysis was conducted, and if multivariate, which adjustment factors were inputted into this. Finally, we extracted individual study results including adjusted and unadjusted odds ratio, risk ratio, Hazard Ratio, HR, mean differences along with their respective confidence intervals, standard errors, and *p*‐values.

### 2.5 Summary measures and synthesis of results

A meta‐analysis was conducted for individual prognostic factors against one of our outcomes if homogenous study methods were utilised, and outcome data was available in ≥3 studies. This required data availability utilising the same definition or categorisation of each prognostic factor, and statistical assessment method to avoid synthesising heterogenous results. Where both univariate and multivariate results were available, the multivariate results were utilised. A random effects analysis was utilised due to presumed heterogeneity using the restricted maximum likelihood approach on a log scale with summary results and confidence intervals back transformed to the original scale. For all analyses Stata 17 software was utilised.

Unfortunately, for most factors and outcomes a meta‐analysis was not feasible due to high variance in assessment methods and prognostic factor definitions. Therefore, for most factors a structured qualitative synthesis was conducted with study results first grouped through the outcome they measured and subsequently through individual prognostic factors, dividing them into patient, oncological and treatment factors. Descriptive statistics were utilised to describe study characteristics. Vote counting of significant results was used to measure the prognostic value of individual factors and direction of effect where more than one study evaluated a specific factor. The effect of publication bias was not evaluated as no outcome presented with 10 or more studies. Study risk of bias rating, certainty of evidence and size of effect were subsequently used to measure the clinical significance of findings.

### 2.6 Study risk of bias and certainty of evidence assessment

The risk of bias of individual studies was assessed by two independent reviewers (NV and JF) with a third reviewer (OB) acting as adjudicator for any discrepancies, using the Quality in Prognostic Studies (QUIPS) tool.^28^ QUIPS was used to assess six domains of potential bias: study participants, study attrition, prognostic factor measurement, outcome measurement, study confounding and statistical analysis and reporting. Each domain was given a rating of low, moderate, or high risk of bias, with an overall rating of bias given for each study. An overall summary table and figure was then created using the robvis tool.^29^ Subsequent assessment of overall certainty of evidence was conducted on a per outcome basis, with the strength of evidence for specific prognostic factors evaluated using the Grading of Recommendations Assessment, Development and Evaluation (GRADE) approach for the assessment of evidence about prognosis.^30^ This was done if two or more studies were available for an individual prognostic factor. GRADE evidence profiles for each outcome and prognostic factor were then created (Supplementary Material S2‐S4).

## 3 RESULTS

### 3.1 Study characteristics

**FIGURE 1:**
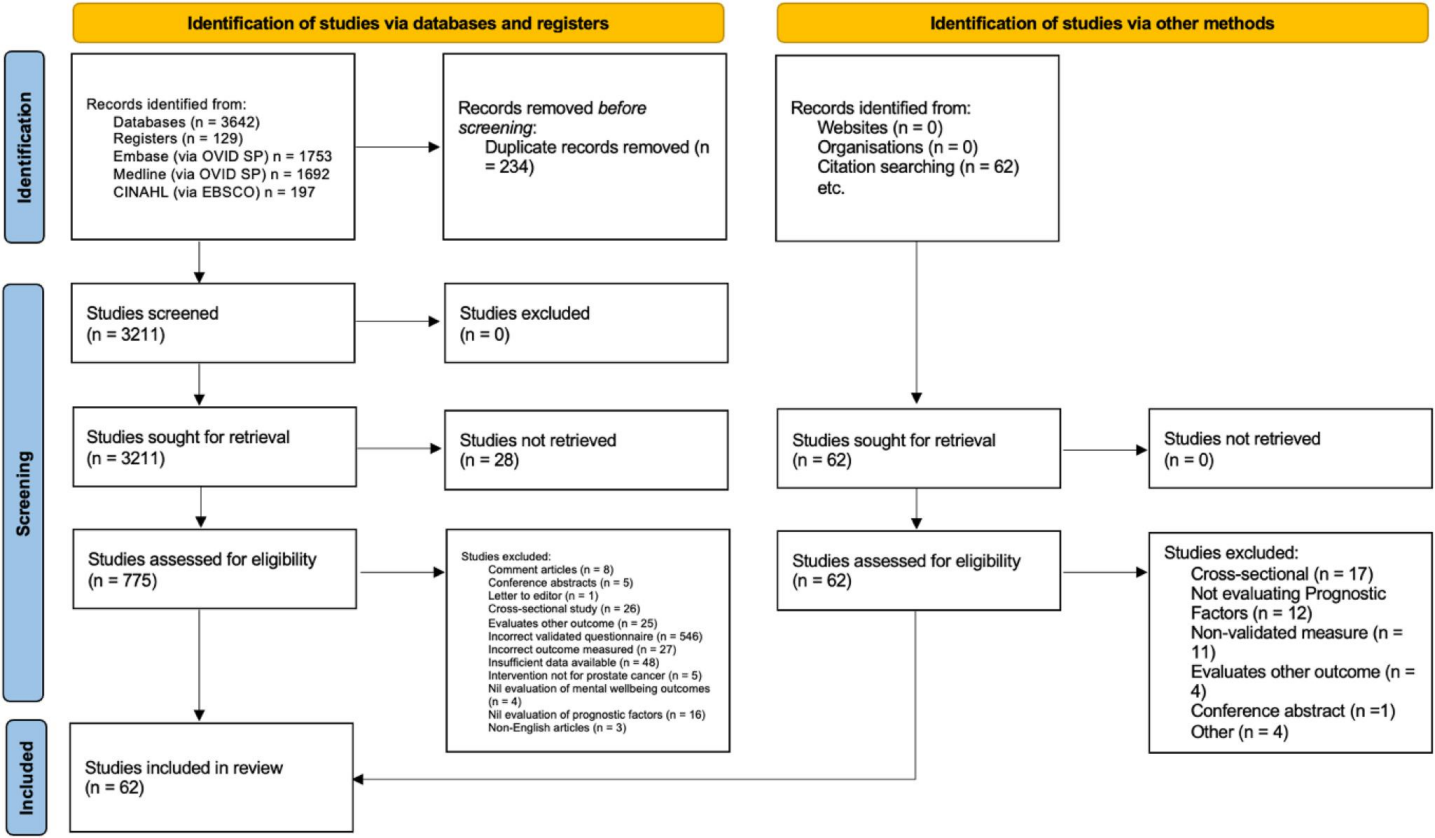
PRISMA flow chart.

A total of 3642 records were identified through the search with 3211 unique studies screened for inclusion. Following title, abstract and full text screen, 62 studies met the inclusion criteria and were included in the final review (Figure 1).

Of included studies, 59 studies were observational and three interventional (Table 1). Most were carried out in the USA (42%), followed by Netherlands (8.1%), Taiwan (8.1%), Australia (6.5%), Italy (6.5%) Canada (6.5%), Sweden (4.9%), UK (3.2%), Germany (3.2%), China (3.2%), Korea (1.6%), Denmark (1.6%), Belgium (1.6%), Brazil (1.6%) and Portugal (1.6%). A total sample size of 291,848 patients was present in these studies, with individual study samples between 37 and 78,552 patients and a median age of 66.9 years. Full references of all included studies are available within Supplementary Material S5.

**TABLE 1:** Summary of study characteristics.

Study	Total *N*. of patients	Age	Treatment received for PCa	Grade, stage or risk classification of cancer
Alvisi 2020	236	64.4	AS	T1‐T2, N0, M0
Boeri 2018	811	Not stated	RP	Not stated
Chen 2020	71	72.5	ADT	Stage I‐IV
Chen 2015	12,872	74	RP, RT, ADT	Not stated
Chien 2018a	117	66.7	RP, RT + ADT, ADT or other	T1‐T4
Chien 2018b	48	67	RP or RT	Stage I‐III
Chung 2017	868	74.1	ADT	Not stated
De Cerqueira 2015	30	64.73	Focal Cryoblation, RT, AS	Gleason <6
Deka 2019	39,965	66.81	RT, RT + ADT	T1‐T3
Dinh 2017	78,552	75.7	ADT	Stage I‐III
Dinh 2016	78,552	75.7	ADT	Stage I‐III
Donovan 2016	1643	62	AS, RP and RT	T1‐T2
Dordoni 2022	823	64	RP, RT, AS	T1‐T2
Dowrick 2018	540	62.2	RP	T1‐T3
Duarte 2022	292	67.8	AS, Curative (RT, RP), pallative (ADT +/− chemotherapy)	T1‐T4, N0‐N1, M0‐M1
Egger 2018	341	69	AS, RP, RT, brachytherapy	T1‐T4
Ene 2006	140	63.1	RP	Stage I‐III
Erim 2019a	1024	Not stated	Not stated	T1‐T3
Erim 2019b	805	Not stated	Not stated	T1‐T3
Ferhava 2021	2445	68.1	ADT	Not stated
Fleshner 2012	302	65.1	AS, ADT	T1‐T2
Friberg 2021	5570	Not stated	RP	Not stated
Gagliano‐Juca 2018	37	67	ADT	Not stated
Hervouet 2013	60	70.04	ADT + RT, RT	Stage I‐III
Hong 2010	584	Not stated	RP	T0–T4
Hoyt 2015	66	65.76	RP or RT	Gleason 6
Hu 2021	194	62.5	RP	T2–T4, N0‐N1
Kohler 2014	329	65.3	RP	T1–T3
Krupski 2005	208	58.98	RP, RT, or ADT	Not stated
Lee 2015	122	67	ADT or RP	Not stated
Lev 2009	159	55.8	RT + ADT, RT, RP	Gleason score 5–10
Luckenbaugh 2022	2742	64	RP, ADT, AS and RT	T1‐T2
Marzouk 2018	463	61	AS	D’Amico low/intermediate risk PC
Mehta 2003	519	64.8	RP, RT, brachytherapy	T1–T4, N1‐N2, M1
Meissner 2021	2417	69.5	RP	Not stated
Mohamed 2012	869	65.45	RT (3D RT, brachytherapy), RP	T1‐T2, N0, M0
Naha 2021	302	65	AS	Not stated
Nordin 2001	99	Not stated	Not stated	T4, N1, M1
Parker 2017	180	67.2	AS	Low risk (Gleason score 3 + 3)
Pearce 2015	195	66.5	AS	<T2a, Gleason <6
Pirl 2008	52	62	ADT	Not stated
Punnen 2013	679	60.1	AS or RP	Not stated
Rosenfeld 2004	341	71.2	RP, RT/Brachytherapy, ADT, AS or Chemotherapy	Localised (T1‐T2), locally advanced (T3‐T4), metastatic (N1‐N3, Ma‐c)
Ruane McAteer 2019	54	62.75	AS or active treatment	Gleason 6 and 7
Sciarra 2018	220	65.3	RP, RT or AS	T1‐T3, N0, M0
Shahinian 2006	50,613	75	ADT	Stage I‐IV
Sharpley 2016	102	71.78	HT	T1‐T4, N0‐N1, M0
Sharpley 2014	1070	67.5	ADT	T2‐T4
Shin 2020	107	Not stated	ADT or RP	Not stated
Steineck 2002	326	64.4	RP, WW	T1‐T2
Tan 2016	119	62.8	AS	Not stated
Tavlaride 2015	350	63.8	RP	T1–T3
Thornton 2012	83	61.83	RP	T1–T3
Timilshina 2012	257	69.1	ADT	T1–T3
Tully 2021	325	Not stated	ADT	Not stated
Van Den Bergh 2010	129	64.6	AS	Non‐palpable, localised
Van Den Bergh 2012	266	65.04	AS, RP, RT, combined	T1‐T2
Van Den Driessche 2016	145	77.8	ADT	Gleason 6–10
Van Stam 2020	434	66.4	AS, ADT + RT	T1‐T2
Van Tol‐Geerdink 2011	288	69	ADT	T1‐T3, N0, M0
Venderbos 2015	150	64.6	AS	Non palpable, localised
Zhang 2017	146	70.4	ADT, RP	T1–T3

Abbreviations: ADT, Androgen Deprivation Therapy; AS, Active Surveillance; PCa, Prostate Cancer RP Radical Prostatectomy; RT, Radiotherapy; TNM, cancer staging (Tumour, Node, Metastasis), Stage I‐III cancer staging; WW, Watchful Waiting.

### 3.2 Depression

Depression was the most investigated outcome across 43 studies with a combined 176,843 patients (Supplementary material S6).

#### 3.2.1 Patient factors

**FIGURE 2:**
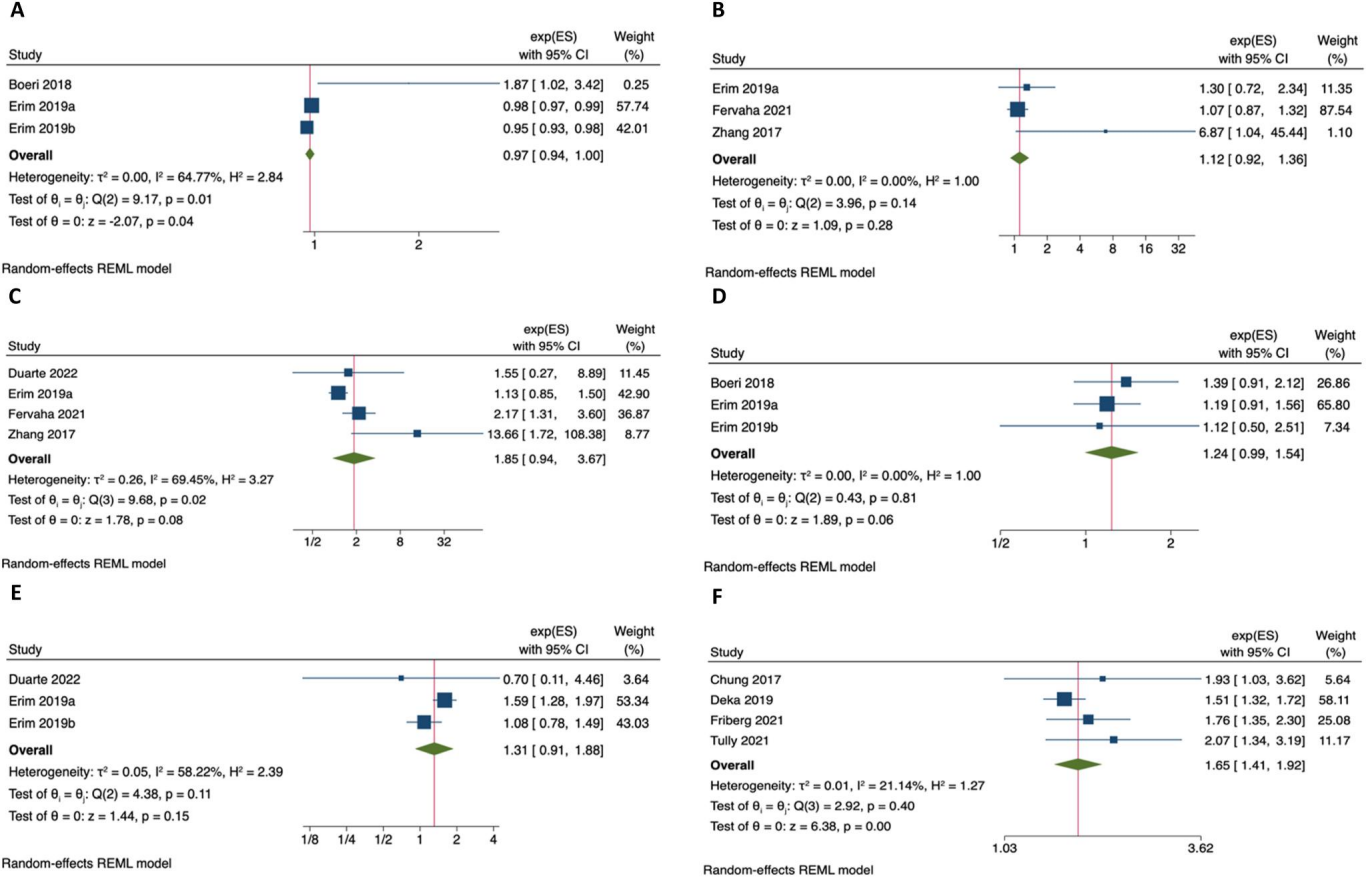
Meta‐analysis of prognostic factors for depression outcomes. (A) Age (Continuous), (B) Alcohol Intake (Yes vs. No), (C) Smoking (Yes vs. Never), (D) Marital Status (Unmarried vs. Married), (E) Comorbidity (Charlson Comorbidity Index Value), (F) Androgen Deprivation Therapy (Given vs. Not).

Age was the most evaluated prognostic factor identified in 13 studies. A pooled analysis of three studies measuring this as a continuous variable (Figure 2) demonstrated increasing age to be associated with a significant reduction in depression (OR 0.97, 95% CI 0.94–1.00, *p* = 0.04). Narrative synthesis of all studies supported this with younger patients being associated with depression post diagnosis consistently with a low degree of certainty of evidence. Alcohol intake was, however, not associated with higher levels of depression when pooling three studies (OR 1.10 95% CI 0.92–1.36, *p* = 0.38), with only a single study out of five identifying a significant association on narrative synthesis (moderate certainty of evidence). Similarly, there was no association when evaluating smoking status across four pooled studies (OR 1.85, 95% CI 0.94–3.67, *p* = 0.08), with only two of six studies identifying current smokers to be associated with higher levels of depression on narrative synthesis with (moderate certainty of evidence).

Being unmarried was associated with higher levels of depression post diagnosis, albeit not quite reaching statistical significance on pooling of three studies (OR 1.24, 95% CI 0.99–1.54, *p* = 0.06), with two of five studies on narrative synthesis demonstrating this (moderate certainty of evidence). When evaluating comorbidities, Charlson Comorbidity Scores were not associated with higher levels of depression (OR 1.31, 95% CI 0.91–1.88, *p* = 0.15); however, a broader narrative analysis with evidence of low certainity of the presence of comorbidities demonstrated more consistently an association in four of eight studies. No other patient factors were able to be pooled for depression.

On a purely narrative synthesis, several other factors appeared to be consistently associated with depression (Table 2). A known mental health diagnosis or a poor baseline status was one of these, seen to be a significant factor across all four studies evaluating this with high certainty of evidence and to be of large effect size (OR 2.44–13.06). Additionally, white ethnicity (high certainty of evidence) and poorer sexual function (moderate certainty of evidence) may also be associated with higher levels of depression. However, there are significantly fewer combined patients across all studies within the non‐white ethnicity group (26,184 patients) compared to those of white ethnicity (94,161 patients) meaning fewer evaluations of other ethinicities were conducted. Importantly, education level did not appear to be associated with higher levels of depression (very low certainty of evidence), with several other exposures exhibiting more heterogeneous or less certain associations including employment and income. Urinary function also did not show much consistent association with depression, with only one of three studies demonstrating a significant relationship with generalised urinary function. Post‐op urinary continence specifically was evaluated in one study, however appeared to not associated depression scores.^31^ Unfortunately, numerous factors were evaluated only in single studies, meaning little formal evaluation of their role could be undertaken, including potentially important factors such as the presence of decisional regret, religion, and other ethnicities.

**TABLE 2:** Summary of narrative prognostic factor synthesis for mental wellbeing outcomes.

Prognostic factor	Outcome	*N*. Patients	Sig. Association with outcome (n. Studies/total studies)	Directionality (number of studies/total significant studies)	Effect size range	Method of assessment
Patient factors						
Age	Depression	137,347	6/13	Younger age increases risk of depression (4/6)	OR – 0.49–1.87	Multivariate – 3
						Univariate – 10
	Anxiety	80,463	3/9	Conflicting	OR – 0.85–1.51	Multivariate – 1
						Univariate – 8
	FCR	3229	0/4	N/A	‐	Multivariate – 0
						Univariate – 4
Ethnicity (black vs. white ethnicity)	Depression	120,346	3/4	Black ethnicity lower risk of depression than white ethnicity (2/3)	OR – 0.63–1.33	Multivariate – 0
						Univariate – 4
	Anxiety	79,229	1/4	Black ethnicity lower risk of anxiety than white ethnicity (1/1)	OR – 0.77	Multivariate – 1
						Univariate – 3
Marital status	Depression	81,412	2/5	Unmarried individuals at increased risk of depression (2/5)	OR – 1.12–1.39	Multivariate – 5
						Univariate – 0
	Anxiety	80,381	2/5	Unmarried individuals at increased risk of anxiety (2/2)	HR – 1.21–1.22	Multivariate – 1
						Univariate – 4
Alcohol	Depression	43,872	1/5	Alcohol use increased risk of depression (1/1)	OR – 1.07–6.87	Multivariate – 0
						Univariate – 5
Smoking	Depression	44,092	2/6	Current smokers at increased risk of depression (2/2)	OR 1.29–1.55	Multivariate – 0
						Univariate – 6
Employment	Depression	4903	3/6	Conflicting	OR – 0.38–1.74	Multivariate – 1
						Univariate – 5
	Anxiety	677	0/4	N/A	OR – 0.67–1.73	Multivariate – 1
						Univariate – 3
Income	Depression	61,104	1/6	Lower income increased risk of depression (1/1)	OR ‐ 1.57	Multivariate – 0
						Univariate – 6
Education level	Depression	44,868	0/7	N/A	OR ‐ 0.22–1.56	Multivariate – 1
						Univariate – 6
	Anxiety	1619	0/6	N/A	OR – 0.41 – 1.82	Multivariate – 1
						Univariate – 5
	FCR	3872	1/4	Lower education status lowered risk of FCR (1/1)	OR – 0.23	Multivariate – 0
						Univariate – 4
BMI	Depression	3064	1/4	Increasing BMI increased risk of depression (1/1)	OR – 1.07–4.15	Multivariate – 1
						Univariate – 3
	Anxiety	631	0/3	N/A	OR – 0.33–0.34	Multivariate – 0
						Univariate – 3
Baseline mental health status	Depression	44,239	4/4	Known diagnosis, poorer baseline status and antidepressant use increases risk of depression (4/4)	OR 2.44–3.67	Multivariate – 0
						Univariate – 4
Co‐morbidities	Depression	142,393	4/8	Increasing co‐morbidities increases risk of depression (4/4)	OR – 0.70–1.87	Multivariate – 0
						Univariate – 8
	Anxiety	79,313	1/4	Increasing co‐morbidities increases risk of anxiety (1/1)	OR – 0.13–2.41	Multivariate – 1
						Univariate ‐ 3
Sexual function	Depression	3700	2/5	Poorer sexual function increases risk of depression (2/2)	OR – 1.53–7.31	Multivariate – 3
						Univariate – 2
Urinary function	Depression	1148	1/3	Poorer urinary function increases risk of depression (1/1)	OR – 0.71	Multivariate – 2
						Univariate – 1
	Anxiety	456	2/3	Poorer urinary function increases risk of anxiety (2/2)	OR – 3.44	Multivariate – 1
						Univariate – 3
Oncological factors						
Cancer stage	Depression	4683	0/5	N/A	OR – 0.64–1.34	Multivariate – 1
						Univariate – 4
	Anxiety	1141	1/5	Increasing stage increases risk of anxiety (1/1)	HR – 0.87–1.02	Multivariate – 1
						Univariate – 4
	FCR	812	0/3	N/A	‐	Multivariate – 1
						Univariate – 2
Gleason grade	Depression	81,324	1/4	Increasing grade increased risk of depression (1/1)	HR – 1.08–1.09	Multivariate – 2
						Univariate – 2
	Anxiety	79,585	1/4	Increasing grade increased risk of anxiety (1/1)	HR – 0.87–1.02	Multivariate – 0
						Univariate – 4
	FCR	1283	0/3	N/A	‐	Multivariate – 1
						Univariate – 2
PSA	Depression	515	1/4	Increasing PSA value increases risk of depression (1/1)	‐	Multivariate – 3
						Univariate – 1
	Anxiety	1108	2/4	Increasing PSA value increases risk of anxiety (2/2)	‐	Multivariate – 1
						Univariate – 3
Time since diagnosis	Depression	3750	6/10	Increasing time since diagnosis reduces risk of depression (4/6)	OR – 0.33–0.73	Multivariate – 1
						Univariate – 9
	Anxiety	2480	6/10	Increasing time since diagnosis reduces risk of anxiety (5/6)	OR – 0.95	Multivariate – 0
						Univariate – 10
	FCR	4019	2/4	Conflicting	OR – 1.10	Multivariate – 0
						Univariate – 4
Treatment factors						
Use of ADT	Depression	192,476	10/15	ADT use increases risk of depression (10/10)	HR ‐ 1.50–2.07	Multivariate – 2
						Univariate – 13
	Anxiety	79,140	2/4	ADT use increases risk of anxiety (2/2)	HR – 1.05–1.16	Multivariate – 1
						Univariate – 3
RP versus RT	Depression	15,604	2/4	Conflicting	HR – 1.53	Multivariate – 0
						Univariate – 4
	Anxiety	2345	0/4	N/A	‐	Multivariate – 0
						Univariate – 4
AS versus Radical treatment	Depression	6267	1/9	Radical treatment increases risk of depression (1/1)	OR – 0.46–0.52	Multivariate – 1
						Univariate – 8
	Anxiety	3959	0/9	N/A	OR – 0.62–1.10	Multivariate – 0
						Univariate – 9

Abbreviations: ADT, Androgen Deprivation Therapy; AS, Active Surveillance; BMI, Body Mass Index; FCR, Fear of Cancer Recurrence; HR, Hazard Ratio; OR, Odds Ratio; PC, Prostate Cancer; PSA, Prostate Specific Antigen; RP, Radical Prostatectomy; RT, Radiotherapy.

#### 3.2.2 Oncological factors

No pooling of any oncological factor results was possible, and few showed any or consistent evidence for effect. Prostate Cancer stage was not seen to be associated with depression after diagnosis in all five studies and 4683 patients evaluating this (very low certainty of evidence). Similarly, Gleason grade (moderate certainty of evidence) and Prostate Specific Antigen (PSA) values (very low certainty of evidence) had little consistent association with depression. However, increasing time since diagnosis which was evaluated across 10 studies was seen to be associated with lower levels of depression (moderate certainty of evidence), with maximum time since diagnosis within these studies at 84 months.

#### 3.2.3 Treatment factors

The use of ADT was consistently reported to be associated with depression in patients with advanced metastatic disease or when used as neo‐adjuvant/adjuvant treatment in those with localised or locally advanced disease (high certainty of evidence). This was seen in 10 of 15 studies evaluating ADT use across 192,476 patients, with pooling of four study results supporting this (HR 1.65, 95% CI 1.41–1.92, *p* < 0.01). However, conflicting evidence for effect was seen when comparing radical treatment modalities of prostatectomy against radiotherapy for localised or locally advanced disease (very low certainty of evidence) with no difference seen between those undergoing Active Surveillance, AS against any radical treatment for low risk localised disease in eight of nine studies (very low certainty of evidence). Few other treatment factors were evaluated consistently including prostatectomy approach or the use of focal treatment options.

### 3.3 Anxiety

Thirty studies were identified evaluating any prognostic factors for anxiety, with a combined sample size of 87,419 patients, although the majority of this cohort arose from a single study of 78,552 patients.^32^ Supplementary Material S7 summarises individual study results for these. No pooling of any prognostic factor for anxiety was possible due to varying analysis methods or exposure categorisation.

#### 3.3.1 Patient factors

The most evaluated patient factor was age across nine studies. However, no consistent association was seen with anxiety in these studies with varying or unclear directionality of effect seen across the three significant studies identified (very low certainty of evidence). Some evidence was seen for unmarried individuals being associated with poorer anxiety outcomes within two of five studies; however, this was only of moderate certainty of evidence. When evaluating ethnicity only a single study of four identified lower anxiety in those of black ethnicity (moderate certainty of evidence), however, as with depression significantly higher number of patients were included overall of white ethnicity (65,796 patients) than of non‐white ethnicities (12,756 patients). Similarly, the presence of comorbidities was seen to be positively associated in one of four studies (low certainty of evidence).

Post radical treatment, urinary function appeared to be an important prognostic factor in two of three studies (high certainty of evidence). These evaluated either mixed incontinence/obstructive urinary symptoms via combined Expanded PC Index Composite (EPIC) scores,^33^, ^34^ or obstructive/irritative symptoms specifically via the International Prostate Symptoms Score.^35^ Obstructive symptoms specifically appeared to have a large effect seen on anxiety symptoms in those with moderate/severe symptoms versus those with mild symptoms in one study of 119 American men undergoing AS (OR 3.44, CI 1.13–10.50).^35^

Factors consistently seen to have no association with anxiety across multiple studies included both education level (very low certainty of evidence), and employment status (very low certainty of evidence). Few other individual factors were consistently evaluated across three or more studies, including the role of sexual dysfunction, religion, other ethnicities, and pre‐existing mental health diagnoses.

#### 3.3.2 Oncological factors

Both cancer stage and Gleason grade demonstrated little association with anxiety with only single studies of five and four total respectively associating increasing stage/grade with anxiety symptoms (both with low certainty of evidence). However, considering PSA values, there appeared to be a possible association with anxiety, but with a very low certainty of evidence. Lastly, time since diagnosis was the most investigated oncological factor in 10 studies combining 2480 patients, with consistent evidence to demonstrate decreasing anxiety with increasing time in five studies, with maximum follow up time within these studies at 2 years (high certainty of evidence).

#### 3.3.3 Treatment factors

The relationship between treatment undergone and subsequent anxiety was investigated in 24 different studies. Receiving ADT was associated with significantly higher anxiety in two of four studies (high certainty of evidence) when evaluating men with advanced/high risk disease. However, more commonly investigated were those undergoing AS against those undergoing any radical treatment across nine studies and 3959 patients, combining individuals with localised or locally advanced disease. Importantly, there was a very consistent demonstration that no differences in anxiety were seen between these groups across all studies identified (very low certainty of evidence). Similarly, no differences were seen in four studies comparing those undergoing prostatectomy and radiotherapy for localised or locally advanced disease with a very low certainty of evidence.

### 3.4 Masculinity

Prognostic factors for masculinity outcomes were not commonly evaluated with only three studies with a total cohort of 1693 patients identified in this review. Each measured masculinity using different scales: EORTIC QLQ‐PR25, The Masculine Self‐Esteem Scale and the Clark health worry and regret scale (Masculinity subscale) (Supplementary Material S8). No individual prognostic factor was evaluated by more than one study. The largest study of 1070 patients by Sharpley et al. however did identify depression‐anxiety to be the most important potential factor when considering a loss of masculinity.^36^ Within this study the term depression‐anxiety was defined by scores from clustered selected items from the EORTC QLQ‐C30 questionnaire chosen to represent DSM‐V criteria for generalised anxiety disorder and major depressive disorder. Few other treatment, oncological or treatment factors were evaluated across these studies.

### 3.5 Body image

Only a single small longitudinal study of 145 patients was identified evaluating prognostic factors for body image issues^37^ (Supplementary Material S9). This largely focussed on the use of ADT on body image disturbance, highlighting its increasing use to be associated body image disturbance. No other patient, oncological or treatment factors were evaluated.

### 3.6 Fear of cancer recurrence

Prognostic factors for FCR as an outcome were evaluated in 10 studies in the review. A total of 5645 patients were assessed using three different diagnostic criteria for FCR (Supplementary Material S10).

#### 3.6.1 Patient factors

Age was not associated with subsequent FCR across all four studies evaluating this factor very low certainty of evidence. Additionally, education level demonstrated little evidence for an association with FCR (moderate certainty of evidence), with only a single study of four demonstrating lower education levels being associated with lower FCR levels. Unfortunately, no other factor was evaluated consistently across three or more studies, meaning that no conclusions could be drawn on the role of ethnicity, employment, pre‐existing mental health status or co‐morbidities on subsequent FCR.

#### 3.6.2 Oncological factors

Both cancer stage and Gleason grade were not seen to be associated with subsequent FCR in any of the three studies evaluating this (both very low certainty of evidence). Interestingly, the role of time since diagnosis was identified to be a significant factor in two of four studies, at up to 31 months post diagnosis. However, there was varying directionality in these, meaning a conflicting evidence base exists surrounding this.

#### 3.6.3 Treatment factors

The role of treatment on subsequent FCR was rarely investigated. Only single studies demonstrated no significant differences between those undergoing radiotherapy and prostatectomy in those with localised or locally advanced disease^33^ or between those undergoing surveillance and prostatectomy for low risk disease.^38^ Whilst individual treatments were not associative, positive surgical margins were seen to be predictive for FCR in a study of 584 prostatectomy patients.^39^

### 3.7 Risk of bias

Overall, risk of bias was low in over half of studies evaluated using QUIPS (52%) (Supplementary Material S11). However, bias due to attrition because of lack of reporting of missing data or patient dropouts scored relatively poorly with two studies scoring a high‐risk bias (3.3%) and 37 studies moderate risk bias (61%). Similarly, bias secondary to confounding was common, with five studies (8.2%) classified as high‐risk bias and 28 as moderate risk bias (46%).

## 4 DISCUSSION

This review synthesised the available evidence on prognostic factors for numerous mental wellbeing outcomes of importance in PC. Whilst few factors had been consistently evaluated in the literature for any given outcome, we identified some which may be of importance. This included the role of younger age, being single, increasing comorbidities and use of ADT in depression, being single, having poorer urinary function, and being closer to the time of diagnosis in anxiety. Of interest was the lack of an association between many oncological factors such as stage and grade with depression, anxiety or FCR. Similarly, outside of the use of ADT, undergoing surveillance versus radical treatment, or prostatectomy against radiotherapy appeared to have little association with these outcomes. These findings may be important, particularly when considering the common conception that those undergoing AS may have increased cancer‐related anxiety as seen in some individual studies.^40^ Lastly, unfortunately very little evidence exists for any prognostic factors when considering body image and masculinity outcomes.

We believe this to be the first review evaluating the broader idea of mental wellbeing outcomes in PC, with limited previous reviews available in cancer patients overall. This is particularly so when considering domains outside of depression and anxiety alone or generic health related quality of life. However, some findings of this review are consistent with other focussed reviews within PC or in other cancer populations. Androgen Deprivation Therapy use for example, has been consistently demonstrated as a risk factor for depression, with a previous meta‐analysis concluding a 41% increased risk of depression with its use, in keeping with the HR of 1.65 identified in our meta‐analysis.^41^ Similarly, when focussing on breast cancer patients a previous review also found younger age and marital status were two important factors in the probability of post‐treatment anxiety.^42^ Lastly, looking at other outcomes such as FCR across broader cancer populations a previous review also demonstrated the lack of a consistent relationship between time and FCR.^43^

### 4.1 Review limitations

While we have identified some potentially important prognostic factors for mental wellbeing outcomes, there are important limitations to consider. Firstly, despite our inclusion criteria selection limiting included studies to longitiduinal studies with only validated outcome evalauation measures, there remains considerable heterogeneity present in the included study populations, population sizes, methods, and predictors evaluation. This was particularly important when evaluating treatment factors, with some variability in the included PC populations with regards to disease stage/risk and follow up durations, making direct comparison across all studies harder to interpret. Additionally, there was variation between assessment methods for the selected outcomes, and whilst all were validated, there is a known variation in the diagnostic accuracies of these tools.^44^ However, most importantly, was the variability in the evaluation of the prognostic factors themselves. There were often varying prognostic factor definitions and dichotomisation of factors, particularly when considering patient factors. This meant that many were only evaluated in single studies, making direct comparison across studies difficult. Additionally, a lack of use of multivariate analysis within most studies means that the independent role of many individual prognostic factors cannot always be certain.

When considering the review itself, limitations include the inability to provide a statistical synthesis for the vast majority of factors and outcomes within the study, largely due to the heterogeneity highlighted previously meaning such a synthesis would not be appropriate. Additionally, this review sought to evaluate five specific mental wellbeing outcomes, these selected as they were seen to be important from previous literature reviews and patient views conducted as background work.^26^ However, mental wellbeing is a broad and dynamic concept with varying definitions of what this constitutes within PC^8^ This meant that other potentially important constructs were not specifically evaluated including distress/cancer‐specific distress, cancer‐specific distress/emotional distress, broader mental health/wellbeing measures (as is used in the Short Form 12 or 36 or other health related quality of life measures), and other mental health diagnoses (such as serious mental illnesses). Many of these were excluded due to their often broad or varying definitions within the literature, overlapping nature with selected outcomes, or reduced frequency within the population of interest.^45^ Therefore, some potentially useful insights on prognostic factors within other concepts were not explored within this review, such as is seen in long term follow up studies evaluating distress or broad mental health.^46^, ^47^ Lastly, as with any review, due to the broad nature of the outcomes and source of the included articles, it is always possible pertinent articles were missed, particularly non‐English language articles.

Future research is needed to expand the current evidence available on prognostic factors for mental wellbeing outcomes in PC and address some of the above limitations. This is particularly true for certain outcomes such as body image and masculinity where there is a real lack of any evidence. These outcomes have been demonstrated to be important for patients' quality of life and therefore also require further attention.^16^ Additionally, several potentially important factors, including treatments undergone, demonstrated wide variation and inconsistency in results and should receive attention. Future studies evaluating these should consist of large longitudinal studies which are powered for these specific analyses, evaluating baseline factors prior to treatment and incorporating more standardised criteria. Lastly, analysis methods require improvement, including greater use of multivariate analysis to identify independent factors of importance, with better transparency in adjustment factor selection. A better understanding of these important prognostic factors through these methods would allow development of models to better risk stratify patients at baseline and target those at high risk early in their pathway who might benefit particularly from preventative and/or interventional care.

### 4.2 Clinical implications

This review does have important clinical implications. Quality of life and mental wellbeing in PC is becoming an increasingly important consideration and is acknowledged to be reduced in PC patients by the European Association of Urology and the American Association of Urology.^11^ However, survivorship care remains unstructured in many aspects of PC follow up.^48^ Prognostic factors identified in this review including age, relationship status, time since diagnosis, and ADT could therefore be incorporated within current routine and survivorship follow up care. This will aid in highlighting groups of patients who may require higher surveillance for mental wellbeing outcomes or those who may benefit from a more targeted intervention to attempt to reduce the occurrence of these outcomes or their subsequent severity once identified. Numerous previous interventions, and prevention strategies have previously been mentioned in the literature including cognitive therapy, mindfulness‐based therapy, exercise, and lower cost technology interventions which have all previously been highlighted as effective measures to improve a plethora of the included outcomes within this review.^49^, ^50^, ^51^, ^52^, ^53^, ^54^ This would therefore allow for a more individualised approach in attempting to improve patient quality of life and wellbeing post diagnosis.

## 5 CONCLUSION

Mental wellbeing issues are common in patients with PC and a knowledge of prognostic factors for their development is important. Key factors we identified included age and co‐morbidities for depression, as well as time since diagnosis and urinary function for anxiety. Importantly, outside of the use of ADT, there appears to be little association between treatment undertaken and these outcomes. However, limited evidence exists for many individual factors for FCR, and no meaningful conclusions were possible when considering body image and masculinity outcomes. Nevertheless, considering the identified prognostic factors within PC follow‐up care is important for the early recognition of these outcomes. This will allow clinicians to provide targeted screening or support to groups of patients who are seen as at‐risk of developing these mental wellbeing issues, thereby attempting to improve patient quality of life.

## AUTHOR CONTRIBUTIONS

Conceptualization: Oliver Brunckhorst, Robert Stewart, Prokar Dasgupta, Kamran Ahmed. Methodology: Neel Vyas, Oliver Brunckhorst, Kamran Ahmed. Formal analysis and investigation: Neel Vyas, Oliver Brunckhorst, Jack B Fanshawe. Writing ‐ original draft preparation: Neel Vyas, Oliver Brunckhorst. Writing ‐ review and editing: Robert Stewart, Prokar Dasgupta, Kamran Ahmed. Funding acquisition: Neel Vyas, Oliver Brunckhorst, Prokar Dasgupta, Kamran Ahmed. Resources: Prokar Dasgupta, Kamran Ahmed. Supervision: Robert Stewart, Prokar Dasgupta, Kamran Ahmed.

## CONFLICT OF INTEREST STATEMENT

Robert Stewart declares research support received in the last 3 years from Janssen, GSK and Takeda. The other authors have no conflicts of interest to declare that are relevant to the content of this article.

## Supporting information

Supporting Information S1

Supporting Information S2

Supporting Information S3

Supporting Information S4

Supporting Information S5

Supporting Information S6

Supporting Information S7

Supporting Information S8

Supporting Information S9

Supporting Information S10

Supporting Information S11

## Data Availability

The data that support the findings of this study are available from the corresponding author upon reasonable request.

## References

[1] Rawla P . Epidemiology of prostate cancer. World J Oncol. 2019;10(2):63‐89. 10.14740/wjon1191 31068988 PMC6497009

[2] Stangelberger A , Waldert M , Djavan B . Prostate cancer in elderly men. Rev Urol. 2008;10(2):111‐119. 18660852 PMC2483315

[3] Trama A , Foschi R , Larrañaga N , et al. Survival of male genital cancers (prostate, testis and penis) in Europe 1999–2007: results from the EUROCARE‐5 study. Eur J Cancer. 2015;51(15):2206‐2216. 10.1016/j.ejca.2015.07.027 26421823

[4] Ito K . Prostate‐specific antigen‐based screening for prostate cancer: evidence, controversies and future perspectives. Int J Urol. 2009;16(5):458‐464. 10.1111/j.1442-2042.2009.02293.x 19341365

[5] Halpern MT , Argenbright KE . Evaluation of effectiveness of survivorship programmes: how to measure success? Lancet Oncol. 2017;18(1):e51‐e59. 10.1016/s1470-2045(16)30563-0 28049577

[6] Richards M , Corner J , Maher J . The National Cancer Survivorship Initiative: new and emerging evidence on the ongoing needs of cancer survivors. Br J Cancer. 2011;105(1):S1‐S4. 10.1038/bjc.2011.416 PMC3251952 22048027

[7] Brunckhorst O , Hashemi S , Martin A , et al. Depression, anxiety, and suicidality in patients with prostate cancer: a systematic review and meta‐analysis of observational studies. Prostate Cancer Prostatic Dis. 2021;24(2):281‐289. 10.1038/s41391-020-00286-0 32978524

[8] Prostate Cancer UK . Research into Wellbeing Services for Men with Prostate Cancer –final Report; 2014. https://prostatecanceruk.org/media/2460122/Report‐Wellbeing‐services‐for‐men‐with‐prostate‐cancer.pdf

[9] Lebel S , Ozakinci G , Humphris G , et al. From normal response to clinical problem: definition and clinical features of fear of cancer recurrence. Support Care Cancer. 2016;24(8):3265‐3268. 10.1007/s00520-016-3272-5 27169703

[10] Johanes C , Monoarfa RA , Ismail RI , Umbas R . Anxiety level of early‐ and late‐stage prostate cancer patients. Prostate Int. 2013;1(4):177‐182. 10.12954/pi.13027 24392443 PMC3879056

[11] James C , Brunckhorst O , Eymech O , Stewart R , Dasgupta P , Ahmed K . Fear of cancer recurrence and PSA anxiety in patients with prostate cancer: a systematic review. Support Care Cancer. 2022;30(7):5577‐5589. 10.1007/s00520-022-06876-z 35106656 PMC9135793

[12] Rhoten BA . Body image disturbance in adults treated for cancer ‐ a concept analysis. J Adv Nurs. 2016;72(5):1001‐1011. 10.1111/jan.12892 26748811

[13] Hearn J . Research in men and masculinities: some sociological issues and possibilities. Aust New Zeal J Sociol. 1994;30(1):47‐70. 10.1177/144078339403000104

[14] Connell RW , Messerschmidt JW . Hegemonic masculinity: rethinking the concept. Gend Soc. 2005;19(6):829‐859. 10.1177/0891243205278639

[15] Wall D , Kristjanson L . Men, culture and hegemonic masculinity: understanding the experience of prostate cancer. Nurs Inq. 2005;12(2):87‐97. 10.1111/j.1440-1800.2005.00258.x 15892724

[16] Bowie J , Brunckhorst O , Stewart R , Dasgupta P , Ahmed K . Body image, self‐esteem, and sense of masculinity in patients with prostate cancer: a qualitative meta‐synthesis. J Cancer Surviv. 2022;16(1):95‐110. 10.1007/s11764-021-01007-9 33963973 PMC8881246

[17] Anderson E , McCormack M . Inclusive Masculinity Theory: overview, reflection and refinement. J Gend Stud. 2018;27(5):547‐561. 10.1080/09589236.2016.1245605

[18] Bowie J , Brunckhorst O , Stewart R , Dasgupta P , Ahmed K . Body image, self‐esteem, and sense of masculinity in patients with prostate cancer: a qualitative meta‐synthesis. J Cancer Surviv. 2022;16(1):95‐110. 10.1007/s11764-021-01007-9 33963973 PMC8881246

[19] Brawer MK . Hormonal therapy for prostate cancer. Rev Urol. 2006;8(Suppl 2):S35‐S47. PMC1578721 17021641

[20] Riley RD , Hayden JA , Steyerberg EW , et al. Prognosis research strategy (PROGRESS) 2: prognostic factor research. PLOS Med. 2013;10(2):e1001380. 10.1371/journal.pmed.1001380 23393429 PMC3564757

[21] Rethlefsen ML , Kirtley S , Waffenschmidt S , et al. PRISMA‐S: an extension to the PRISMA statement for reporting literature searches in systematic reviews. Syst Rev. 2021;10(1):39. 10.5195/jmla.2021.962 33499930 PMC7839230

[22] Page MJ , McKenzie JE , Bossuyt PM , et al. The PRISMA 2020 statement: an updated guideline for reporting systematic reviews. BMJ. 2021;372:n71. 10.1136/bmj.n71 33782057 PMC8005924

[23] Campbell M , McKenzie JE , Sowden A , et al. Synthesis without meta‐analysis (SWiM) in systematic reviews: reporting guideline. BMJ. 2020;368:l6890. 10.1136/bmj.l6890 31948937 PMC7190266

[24] Riley RD , Moons KGM , Snell KIE , et al. A guide to systematic review and meta‐analysis of prognostic factor studies. BMJ. 2019;364:k4597. 10.1136/bmj.k4597 30700442

[25] Eymech O , Brunckhorst O , Fox L , et al. An exploration of wellbeing in men diagnosed with prostate cancer undergoing active surveillance: a qualitative study. Support Care Cancer. 2022;30(6):5459‐5468. 10.1007/s00520-022-06976-w 35304633 PMC8933126

[26] Vyas N , Brunckhorst O , Fox L , et al. Undergoing radical treatment for prostate cancer and its impact on wellbeing: a qualitative study exploring men's experiences. PLoS One. 2022;17(12):e0279250. 10.1371/journal.pone.0279250 36525457 PMC9757548

[27] Ouzzani M , Hammady H , Fedorowicz Z , Elmagarmid A . Rayyan‐a web and mobile app for systematic reviews. Syst Rev. 2016;5(1):210. 10.1186/s13643-016-0384-4 27919275 PMC5139140

[28] Grooten WJA , Tseli E , Äng BO , et al. Elaborating on the assessment of the risk of bias in prognostic studies in pain rehabilitation using QUIPS—aspects of interrater agreement. Diagnostic Prognostic Res. 2019;3(1):5. 10.1186/s41512-019-0050-0 PMC6460536 31093575

[29] McGuinness LA , Higgins JPT . Risk‐of‐bias VISualization (robvis): an R package and Shiny web app for visualizing risk‐of‐bias assessments. Res Synth Methods. 2021;12(1):55‐61. 10.1002/jrsm.1411 32336025

[30] Foroutan F , Guyatt G , Zuk V , et al. GRADE Guidelines 28: use of GRADE for the assessment of evidence about prognostic factors: rating certainty in identification of groups of patients with different absolute risks. J Clin Epidemiol. 2020;121:62‐70. 10.1016/j.jclinepi.2019.12.023 31982539

[31] Boeri L , Capogrosso P , Ventimiglia E , et al. Depressive symptoms and low sexual desire after radical prostatectomy: early and long‐term outcomes in a real‐life setting. J Urol. 2018;199(2):474‐480. 10.1016/j.juro.2017.08.104 28867561

[32] Dinh KT , Reznor G , Muralidhar V , et al. Association of androgen deprivation therapy with depression in localized prostate cancer. J Clin Oncol. 2016;34(16):1905‐1912. 10.1200/jco.2015.64.1969 27069075 PMC4966343

[33] Chien CH , Chuang CK , Liu KL , Wu CT , Pang ST , Chang YH . Positive and negative affect and prostate cancer‐specific anxiety in Taiwanese patients and their partners. Eur J Oncol Nurs. 2018;37:1‐11. 10.1016/j.ejon.2018.09.004 30473044

[34] Chien CH , Chuang CK , Liu KL , et al. Effects of individual and partner factors on anxiety and depression in Taiwanese prostate cancer patients: a longitudinal study. Eur J Cancer Care. 2018;27(2):e12753. 10.1111/ecc.12753 28921733

[35] Tan HJ , Marks LS , Hoyt MA , et al. The relationship between intolerance of uncertainty and anxiety in men on active surveillance for prostate cancer. J Urol. 2016;195(6):1724‐1730. 10.1016/j.juro.2016.01.108 26872841 PMC4871722

[36] Sharpley CF , Bitsika V , Denham JW . Factors associated with feelings of loss of masculinity in men with prostate cancer in the RADAR trial. Psycho Oncol. 2014;23(5):524‐530. 10.1002/pon.3448 24829952

[37] van den Driessche H , Mattelaer P , van Oyen P , et al. Changes in body image in patients with prostate cancer over 2 Years of treatment with a gonadotropin‐releasing hormone analogue (triptorelin): results from a Belgian non‐interventional study. Drugs Real World Outcomes. 2016;3(2):183‐190. 10.1007/s40801-016-0074-5 27398297 PMC4914546

[38] Egger SJ , Calopedos RJ , O'Connell DL , Chambers SK , Woo HH , Smith DP . Long‐term psychological and quality‐of‐life effects of active surveillance and watchful waiting after diagnosis of low‐risk localised prostate cancer. Eur Urol. 2018;73(6):859‐867. 10.1016/j.eururo.2017.08.013 28851582

[39] Hong YM , Hu JC , Paciorek AT , Knight SJ , Carroll PR . Impact of radical prostatectomy positive surgical margins on fear of cancer recurrence: results from CaPSURE. Urol Oncol. 2010;28(3):268‐273. 10.1016/j.urolonc.2008.07.004 18848785

[40] Klotz L . Active surveillance, quality of life, and cancer‐related anxiety. Eur Urol. 2013;64(1):37‐39. 10.1016/j.eururo.2013.01.023 23433806

[41] Nead KT , Sinha S , Yang DD , Nguyen PL . Association of androgen deprivation therapy and depression in the treatment of prostate cancer: a systematic review and meta‐analysis. Urol Oncol. 2017;35(11):664.e661‐664.e669. 10.1016/j.urolonc.2017.07.016 28803700

[42] Harris J , Cornelius V , Ream E , Cheevers K , Armes J . Anxiety after completion of treatment for early‐stage breast cancer: a systematic review to identify candidate predictors and evaluate multivariable model development. Support Care Cancer. 2017;25(7):2321‐2333. 10.1007/s00520-017-3688-6 28405845 PMC5445146

[43] Koch L , Jansen L , Brenner H , Arndt V . Fear of recurrence and disease progression in long‐term (≥5 years) cancer survivors—a systematic review of quantitative studies. Psycho‐Oncology. 2013;22(1):1‐11. 10.1002/pon.3022 22232030

[44] de Joode JW , van Dijk SEM , Walburg FS , et al. Diagnostic accuracy of depression questionnaires in adult patients with diabetes: a systematic review and meta‐analysis. PLoS One. 2019;14(6):e0218512. 10.1371/journal.pone.0218512 31220131 PMC6586329

[45] Viertiö S , Kiviruusu O , Piirtola M , et al. Factors contributing to psychological distress in the working population, with a special reference to gender difference. BMC Publ Health. 2021;21(1):611. 10.1186/s12889-021-10560-y PMC8006634 33781240

[46] Ralph N , Ng SK , Zajdlewicz L , et al. Ten‐year quality of life outcomes in men with prostate cancer. Psycho Oncol. 2020;29(2):444‐449. 10.1002/pon.5255 31680363

[47] Chambers SK , Ng SK , Baade P , et al. Trajectories of quality of life, life satisfaction, and psychological adjustment after prostate cancer. Psycho Oncol. 2017;26(10):1576‐1585. 10.1002/pon.4342 PMC5655930 27943512

[48] MacAskill F , Shabbir M , Sahai A . Survivorship in prostate cancer following robotic assisted radical prostatectomy‐the time to act is now! Prostate Cancer Prostatic Dis; 2022. 10.1038/s41391-022-00589-4 PMC10876470 36065059

[49] Langelier DM , D'Silva A , Shank J , Grant C , Bridel W , Culos‐Reed SN . Exercise interventions and their effect on masculinity, body image, and personal identity in prostate cancer‐A systematic qualitative review. Psycho Oncol. 2019;28(6):1184‐1196. 10.1002/pon.5060 30875710

[50] Nnate DA , Anyachukwu CC , Igwe SE , Abaraogu UO . Mindfulness‐based interventions for psychological wellbeing and quality of life in men with prostate cancer: a systematic review and meta‐analysis. Psycho Oncol. 2021;30(10):1680‐1690. 10.1002/pon.5749 34139035

[51] Qan'ir Y , Song L . Systematic review of technology‐based interventions to improve anxiety, depression, and health‐related quality of life among patients with prostate cancer. Psycho Oncol. 2019;28(8):1601‐1613. 10.1002/pon.5158 PMC7465427 31222956

[52] Qiu S , Yang L , Deng L , et al. Psychosocial interventions to improve quality of life for men with prostate cancer: a network meta‐analysis of 31 randomised controlled trials. Lancet. 2017;390:S103. 10.1016/s0140-6736(17)33241-5

[53] Bergerot CD , Philip EJ , Bergerot PG , Siddiq N , Tinianov S , Lustberg M . Fear of cancer recurrence or progression: what is it and what can we do about it? Am Soc Clin Oncol Educ Book. 2022;42:1‐10. 10.1200/edbk_100031 35561298

[54] Kang DW , Fairey AS , Boulé NG , Field CJ , Wharton SA , Courneya KS . A randomized trial of the effects of exercise on anxiety, fear of cancer progression and quality of life in prostate cancer patients on active surveillance. J Urol. 2022;207(4):814‐822. 10.1097/ju.0000000000002334 35179044

