# Supplementary material for: Prognostic factors for mental wellbeing in prostate cancer: A systematic review and meta‐analysis
Source: Psychooncology. 2023 Oct 3;32(11):1644–59. doi: 10.1002/pon.6225 (PMC10946963; doi:10.1002/pon.6225)
Supplement: Supplementary file 1 — Supporting Information S1 [file PON-32-1644-s011.docx]

**Supplementary Material 1: Full Search Strategy for Individual Databases**

Embase and MEDLINE via OvidSp

Embase <1974 to 2022 August 16>

Ovid MEDLINE(R) ALL <1946 to August 17, 2022>

1 exp Prostate/ 94837

2 cancer.mp. or exp Neoplasms/ 10169610

3 prostate cancer.mp. or exp Prostatic Neoplasms/ 473259

4 exp Carcinoma/ 2025328

5 malignant.mp. 1128363

6 2 or 4 or 5 10333405

7 1 and 6 55491

8 3 or 7 485577

9 exp Depression/ 701981

10 exp Depressive Disorder/ or low mood.mp. 679529

11 exp Test Anxiety Scale/ or exp Anxiety/ or exp Anxiety, Castration/ or exp Anxiety Disorders/ or exp Test Anxiety/ 701717

12 9 or 10 807440

13 exp Identification, Psychological/ or exp Sexuality/ or exp Gender Identity/ or exp Masculinity/ or exp Self Concept/ 684051

14 fear of recurrence.mp. 1705

15 fear of cancer recurrence.mp. 1377

16 PSA anxiety.mp. 38

17 exp Anxiety/ or prostate cancer related worry.mp. 362708

18 prostate cancer related distress.mp. 0

19 14 or 15 or 16 or 17 or 18 364787

20 exp Body Image/ 44579

21 exp Self Concept/ or self image.mp. 345264

22 body conscious.mp. 23

23 self conscious.mp. 1544

24 prediction.mp. 1035413

25 predictive factor.mp. 27611

26 prognostic factor.mp. or exp Prognosis/ 2755180

27 outcome measure.mp. 225725

28 exp "Quality of Life"/ 840582

29 20 or 21 or 22 or 23 or 28 1155987

30 24 or 25 or 26 or 27 3883664

31 11 or 12 or 13 or 19 or 29 2569092

32 8 and 30 and 31 3445

CINAHL via EBSCO
