# Supplementary material for: Prognostic factors for mental wellbeing in prostate cancer: A systematic review and meta‐analysis
Source: Psychooncology. 2023 Oct 3;32(11):1644–59. doi: 10.1002/pon.6225 (PMC10946963; doi:10.1002/pon.6225)
Supplement: Supplementary file 2 — Supporting Information S2 [file PON-32-1644-s010.docx]

**Supplementary Material 2: GRADE assessment for Prognostic factors for Depression**

| **№ of studies** | **Certainty assessment** | | | | | | **Effect** | | **Certainty** |
| --- | --- | --- | --- | --- | --- | --- | --- | --- | --- |
|  | **Study design** | **Risk of bias** | **Inconsistency** | **Indirectness** | **Imprecision** | **Other considerations** | **№ of individuals** | **Prognostic effect (OR unless specified)** |  |
| Age | | | | | | | | | |
| 13 | observational studies | not serious | serious | not serious | serious | none | 137 347 | 0.49-1.87 | ⨁⨁◯◯ Low |
| Ethnicity (Black vs White Ethnicity) | | | | | | | | | |
| 4 | observational studies | not serious | not serious | not serious | not serious | none | 120 346 | 0.63-1.33 | ⨁⨁⨁⨁ High |
| Marital Status | | | | | | | | | |
| 5 | observational studies | not serious | not serious | not serious | serious | none | 81 412 | 1.12-1.39 | ⨁⨁⨁◯ Moderate |
| Alcohol | | | | | | | | | |
| 5 | observational studies | not serious | serious | not serious | not serious | none | 43 872 | 1.07-6.87 | ⨁⨁⨁◯ Moderate |
| Smoking | | | | | | | | | |
| 6 | observational studies | not serious | serious | not serious | not serious | none | 44 092 | 1.29-1.55 | ⨁⨁⨁◯ Moderate |
| Employment | | | | | | | | | |
| 6 | observational studies | not serious | serious | serious | serious | none | 4 903 | 0.38-1.74 | ⨁⨁◯◯ Low |
| Income | | | | | | | | | |
| 6 | Observational studies | Not serious | serious | Not serious | serious | none | 61 104 | 1.57 | ⨁⨁◯◯ Low |
| Education Level | | | | | | | | | |
| 7 | observational studies | not serious | serious | serious | serious | none | 44 868 | 0.22-1.56 | ⨁◯◯◯ Very low |
| BMI | | | | | | | | | |
| 4 | Observational studies | Not serious | serious | Not serious | Not serious | none | 3 064 | 1.07-4.15 | ⨁⨁⨁◯ Moderate |
| Baseline Mental Health Status | | | | | | | | | |
| 4 | observational studies | not serious | not serious | not serious | not serious | none | 44 239 | 2.44-3.67 | ⨁⨁⨁⨁ High |
| Co-morbidities | | | | | | | | | |
| 8 | Observational studies | Not serious | serious | not serious | serious | none | 142 393 | 0.70-1.87 | ⨁⨁◯◯ Low |
| Sexual Function | | | | | | | | | |
| 5 | observational studies | not serious | serious | not serious | not serious |  | 3 700 | 1.53-7.31 | ⨁⨁⨁◯ Moderate |
| Urinary Function | | | | | | | | | |
| 3 | Observational studies | not serious | serious | not serious | not serious |  | 1 148 | 0.71 | ⨁⨁⨁◯ Moderate |
| Cancer Stage | | | | | | | | | |
| 5 | observational studies | not serious | very serious | very serious | serious | none | 4 683 | HR 0.64-1.34 | ⨁◯◯◯ Very low |
| Gleason Grade | | | | | | | | | |
| 4 | Observational studies | Not serious | serious | Not serious | Not serious | none | 81 324 | HR 1.08-1.09 | ⨁⨁⨁◯ Moderate |
| PSA | | | | | | | | | |
| 4 | observational studies | not serious | serious | not serious | very serious | none | 515 | - | ⨁◯◯◯ Very low |
| Time since diagnosis | | | | | | | | | |
| 10 | Observational studies | Not serious | serious | Not serious | Not serious | none | 3 750 | 0.33-0.73 | ⨁⨁⨁◯ Moderate |
| Use of ADT | | | | | | | | | |
| 15 | observational studies | not serious | not serious | not serious | not serious | none | 192 476 | HR 1.50-2.07 | ⨁⨁⨁⨁ High |
| RP vs RT | | | | | | | | | |
| 4 | Observational studies | Not serious | serious | Very serious | serious | none | 15 604 | HR 1.53 | ⨁◯◯◯ Very low |
| AS vs Radical Treatment | | | | | | | | | |
| 9 | observational studies | not serious | very serious | very serious | serious | none | 6 267 | 0.46-0.52 | ⨁◯◯◯ Very low |
