# Supplementary material for: Prognostic factors for mental wellbeing in prostate cancer: A systematic review and meta‐analysis
Source: Psychooncology. 2023 Oct 3;32(11):1644–59. doi: 10.1002/pon.6225 (PMC10946963; doi:10.1002/pon.6225)
Supplement: Supplementary file 3 — Supporting Information S3 [file PON-32-1644-s003.docx]

**Supplementary Material 3: GRADE assessment for Prognostic factors for Anxiety**

| **№ of studies** | **Certainty assessment** | | | | | | **Effect** | | **Certainty** |
| --- | --- | --- | --- | --- | --- | --- | --- | --- | --- |
|  | **Study design** | **Risk of bias** | **Inconsistency** | **Indirectness** | **Imprecision** | **Other considerations** | **№ of individuals** | **Prognostic effect (OR unless specified)** |  |
| Age | | | | | | | | | |
| 9 | observational studies | not serious | serious | very serious | serious | none | 80 463 | 0.85-1.51 | ⨁◯◯◯ Very low |
| Ethnicity (Black vs White Ethnicity) | | | | | | | | | |
| 4 | observational studies | not serious | serious | not serious | not serious | none | 79 229 | 0.77 | ⨁⨁⨁◯ Moderate |
| Marital Status | | | | | | | | | |
| 5 | observational studies | not serious | serious | not serious | not serious | none | 80 381 | 1.21-1.22 | ⨁⨁⨁◯ Moderate |
| Employment | | | | | | | | | |
| 4 | observational studies | not serious | very serious | very serious | serious | none | 677 | 0.67-1.73 | ⨁◯◯◯ Very low |
| Education Level | | | | | | | | | |
| 6 | observational studies | not serious | very serious | very serious | serious | none | 1 619 | 0.41-1.82 | ⨁◯◯◯ Very low |
| BMI | | | | | | | | | |
| 3 | Observational studies | Not serious | serious | Not serious | Not serious | none | 631 | 0.33-0.34 | ⨁⨁⨁◯ Moderate |
| Co-morbidities | | | | | | | | | |
| 4 | Observational studies | Not serious | serious | not serious | serious | none | 79 313 | 0.13-2.41 | ⨁⨁◯◯ Low |
| Urinary Function | | | | | | | | | |
| 3 | Observational studies | not serious | Not serious | not serious | not serious | none | 456 | 3.44 | ⨁⨁⨁⨁ High |
| Cancer Stage | | | | | | | | | |
| 5 | observational studies | not serious | serious | Not serious | serious | none | 1 141 | HR 0.87-1.02 | ⨁⨁◯◯ Low |
| Gleason Grade | | | | | | | | | |
| 4 | Observational studies | Not serious | serious | Not serious | serious | none | 79 585 | HR 0.87-1.02 | ⨁⨁◯◯ Low |
| PSA | | | | | | | | | |
| 4 | observational studies | not serious | serious | not serious | very serious | none | 1 108 | - | ⨁◯◯◯ Very low |
| Time since diagnosis | | | | | | | | | |
| 10 | Observational studies | Not serious | Not serious | Not serious | Not serious | none | 2 480 | 0.95 | ⨁⨁⨁⨁ High |
| Use of ADT | | | | | | | | | |
| 4 | observational studies | not serious | not serious | not serious | not serious | none | 79 140 | HR 1.05-1.16 | ⨁⨁⨁⨁ High |
| RP vs RT | | | | | | | | | |
| 4 | Observational studies | Not serious | Very serious | Very serious | very serious | none | 2 345 | - | ⨁◯◯◯ Very low |
| AS vs Radical Treatment | | | | | | | | | |
| 9 | observational studies | not serious | very serious | very serious | serious | none | 3 959 | 0.62-1.10 | ⨁◯◯◯ Very low |
