# Supplementary material for: Prognostic factors for mental wellbeing in prostate cancer: A systematic review and meta‐analysis
Source: Psychooncology. 2023 Oct 3;32(11):1644–59. doi: 10.1002/pon.6225 (PMC10946963; doi:10.1002/pon.6225)
Supplement: Supplementary file 4 — Supporting Information S4 [file PON-32-1644-s002.docx]

**Supplementary Material 4: GRADE assessment for Prognostic factors for Fear of Cancer Recurrence**

| **№ of studies** | **Certainty assessment** | | | | | | **Effect** | | **Certainty** |
| --- | --- | --- | --- | --- | --- | --- | --- | --- | --- |
|  | **Study design** | **Risk of bias** | **Inconsistency** | **Indirectness** | **Imprecision** | **Other considerations** | **№ of individuals** | **Prognostic effect (OR unless specified)** |  |
| Age | | | | | | | | | |
| 4 | observational studies | not serious | Very serious | very serious | Very serious | none | 3 229 | - | ⨁◯◯◯ Very low |
| Education Level | | | | | | | | | |
| 4 | observational studies | not serious | serious | not serious | not serious | none | 3 872 | 0.23 | ⨁⨁⨁◯ Moderate |
| Cancer stage | | | | | | | | | |
| 3 | observational studies | not serious | Very serious | very serious | very serious | none | 812 | - | ⨁◯◯◯ Very low |
| Gleason Grade | | | | | | | | | |
| 3 | observational studies | not serious | very serious | very serious | Very serious | none | 1 283 | - | ⨁◯◯◯ Very low |
| Time Since Diagnosis | | | | | | | | | |
| 4 | observational studies | not serious | serious | very serious | serious | none | 4 019 | 1.10 | ⨁◯◯◯ Very low |
