# Supplementary material for: Prognostic factors for mental wellbeing in prostate cancer: A systematic review and meta‐analysis
Source: Psychooncology. 2023 Oct 3;32(11):1644–59. doi: 10.1002/pon.6225 (PMC10946963; doi:10.1002/pon.6225)
Supplement: Supplementary file 5 — Supporting Information S5 [file PON-32-1644-s009.docx]

**Supplementary Material 5: eReferences**

1. Alvisi MF, Dordoni P, Rancati T, et al. Supporting Patients With Untreated Prostate Cancer on Active Surveillance: What Causes an Increase in Anxiety During the First 10 Months? *Front Psychol.* 2020;11:576459.

2. Boeri L, Capogrosso P, Ventimiglia E, et al. Depressive Symptoms and Low Sexual Desire after Radical Prostatectomy: Early and Long-Term Outcomes in a Real-Life Setting. *J Urol.* 2018;199(2):474-480.

3. Chen PM, Chen SC, Liu CJ, et al. The association between prostate cancer and mood disorders: a nationwide population-based study in Taiwan. *Int Psychogeriatr.* 2015;27(3):481-490.

4. Chen YZ, Chiang PK, Lin WR, et al. The relationship between androgen deprivation therapy and depression symptoms in patients with prostate cancer. *Aging Male.* 2020;23(5):629-634.

5. Chien CH, Chuang CK, Liu KL, Wu CT, Pang ST, Chang YH. Positive and negative affect and prostate cancer-specific anxiety in Taiwanese patients and their partners. *Eur J Oncol Nurs.* 2018;37:1-11.

6. Chien CH, Chuang CK, Liu KL, et al. Effects of individual and partner factors on anxiety and depression in Taiwanese prostate cancer patients: A longitudinal study. *Eur J Cancer Care (Engl).* 2018;27(2):e12753.

7. Chung SD, Kao LT, Lin HC, Xirasagar S, Huang CC, Lee HC. Patients receiving androgen deprivation therapy for prostate cancer have an increased risk of depressive disorder. *PLoS One.* 2017;12(3):e0173266.

8. de Cerqueira MA, Laranja WW, Sanches BC, Monti CR, Reis LO. Burden of focal cryoablation versus brachytherapy versus active surveillance in the treatment of very low-risk prostate cancer: a preliminary head-to-head comprehensive assessment. *Eur J Cancer Care (Engl).* 2015;24(6):929-937.

9. Deka R, Rose BS, Bryant AK, et al. Androgen deprivation therapy and depression in men with prostate cancer treated with definitive radiation therapy. *Cancer.* 2019;125(7):1070-1080.

10. Dinh KT, Reznor G, Muralidhar V, et al. Association of Androgen Deprivation Therapy With Depression in Localized Prostate Cancer. *J Clin Oncol.* 2016;34(16):1905-1912.

11. Dinh KT, Yang DD, Nead KT, Reznor G, Trinh QD, Nguyen PL. Association between androgen deprivation therapy and anxiety among 78 000 patients with localized prostate cancer. *Int J Urol.* 2017;24(10):743-748.

12. Donovan JL, Hamdy FC, Lane JA, et al. Patient-Reported Outcomes after Monitoring, Surgery, or Radiotherapy for Prostate Cancer. *New England Journal of Medicine.* 2016;375(15):1425-1437.

13. Dordoni P, Remmers S, Valdagni R, et al. Cross-cultural differences in men on active surveillance' anxiety: a longitudinal comparison between Italian and Dutch patients from the Prostate cancer Research International Active Surveillance study. *BMC Urol.* 2022;22(1):110.

14. Dowrick AS, Wootten AC, Botti M. Does partnership status affect the quality of life of men having robotic-assisted radical prostatectomy (RARP) for localised prostate cancer? *Appl Nurs Res.* 2018;42:51-55.

15. Duarte V, Araújo N, Lopes C, et al. Anxiety and Depression in Patients with Prostate Cancer, at Cancer Diagnosis and after a One-Year Follow-Up. *Int J Environ Res Public Health.* 2022;19(15).

16. Egger SJ, Calopedos RJ, O'Connell DL, Chambers SK, Woo HH, Smith DP. Long-term Psychological and Quality-of-life Effects of Active Surveillance and Watchful Waiting After Diagnosis of Low-risk Localised Prostate Cancer. *Eur Urol.* 2018;73(6):859-867.

17. Ene KW, Nordberg G, Johansson FG, Sjöström B. Pain, psychological distress and health-related quality of life at baseline and 3 months after radical prostatectomy. *BMC Nursing.* 2006;5(1):8.

18. Erim DO, Bensen JT, Mohler JL, et al. Prevalence and predictors of probable depression in prostate cancer survivors. *Cancer.* 2019;125(19):3418-3427.

19. Erim DO, Bensen JT, Mohler JL, et al. Patterns and predictors of self-reported clinical diagnosis and treatment for depression in prostate cancer survivors. *Cancer Med.* 2019;8(8):3648-3658.

20. Fervaha G, Izard JP, Tripp DA, et al. Psychological morbidity associated with prostate cancer: Rates and predictors of depression in the RADICAL PC study. *Can Urol Assoc J.* 2021;15(6):181-186.

21. Fleshner NE, Lucia MS, Egerdie B, et al. Dutasteride in localised prostate cancer management: the REDEEM randomised, double-blind, placebo-controlled trial. *Lancet.* 2012;379(9821):1103-1111.

22. Friberg AS, Dalton SO, Larsen SB, et al. Risk of Depression After Radical Prostatectomy-A Nationwide Registry-based Study. *Eur Urol Oncol.* 2021;4(4):601-608.

23. Gagliano-Jucá T, Travison TG, Nguyen PL, et al. Effects of Androgen Deprivation Therapy on Pain Perception, Quality of Life, and Depression in Men With Prostate Cancer. *J Pain Symptom Manage.* 2018;55(2):307-317.e301.

24. Hervouet S, Savard J, Ivers H, Savard MH. Depression and androgen deprivation therapy for prostate cancer: a prospective controlled study. *Health Psychol.* 2013;32(6):675-684.

25. Hong YM, Hu JC, Paciorek AT, Knight SJ, Carroll PR. Impact of radical prostatectomy positive surgical margins on fear of cancer recurrence: results from CaPSURE. *Urol Oncol.* 2010;28(3):268-273.

26. Hoyt MA, Carpenter KM. Sexual self-schema and depressive symptoms after prostate cancer. *Psychooncology.* 2015;24(4):395-401.

27. Hu S, Li L, Wu X, Liu Z, Fu A. Post-surgery anxiety and depression in prostate cancer patients: prevalence, longitudinal progression, and their correlations with survival profiles during a 3-year follow-up. *Ir J Med Sci.* 2021;190(4):1363-1372.

28. Köhler N, Friedrich M, Gansera L, et al. Psychological distress and adjustment to disease in patients before and after radical prostatectomy. Results of a prospective multi-centre study. *Eur J Cancer Care (Engl).* 2014;23(6):795-802.

29. Krupski TL, Sonn G, Kwan L, Maliski S, Fink A, Litwin MS. Ethnic variation in health-related quality of life among low-income men with prostate cancer. *Ethn Dis.* 2005;15(3):461-468.

30. Lee M, Jim HS, Fishman M, et al. Depressive symptomatology in men receiving androgen deprivation therapy for prostate cancer: a controlled comparison. *Psychooncology.* 2015;24(4):472-477.

31. Lev EL, Eller LS, Gejerman G, et al. Quality of life of men treated for localized prostate cancer: outcomes at 6 and 12 months. *Support Care Cancer.* 2009;17(5):509-517.

32. Luckenbaugh AN, Wallis CJD, Huang L-C, et al. Association between Treatment for Localized Prostate Cancer and Mental Health Outcomes. *Journal of Urology.* 2022;207(5):1029-1037.

33. Marzouk K, Assel M, Ehdaie B, Vickers A. Long-Term Cancer Specific Anxiety in Men Undergoing Active Surveillance of Prostate Cancer: Findings from a Large Prospective Cohort. *J Urol.* 2018;200(6):1250-1255.

34. Mehta SS, Lubeck D, Pasta DJ, Litwin MS. Fear of cancer recurrence in patients undergoing definitive treatment for prostate cancer: results from CaPSURE. *J Urol.* 2003;170(5):1931-1933.

35. Meissner VH, Olze L, Schiele S, et al. Fear of cancer recurrence and disease progression in long-term prostate cancer survivors after radical prostatectomy: A longitudinal study. *Cancer.* 2021;127(22):4287-4295.

36. Mohamed NE, Bovbjerg DH, Montgomery GH, Hall SJ, Diefenbach MA. Pretreatment depressive symptoms and treatment modality predict post-treatment disease-specific quality of life among patients with localized prostate cancer. *Urol Oncol.* 2012;30(6):804-812.

37. Naha U, Freedland SJ, Abern MR, Moreira DM. The association of cancer-specific anxiety with disease aggressiveness in men on active surveillance of prostate cancer. *Prostate Cancer Prostatic Dis.* 2021;24(2):335-340.

38. Nordin K, Berglund G, Glimelius B, Sjödén PO. Predicting anxiety and depression among cancer patients: a clinical model. *Eur J Cancer.* 2001;37(3):376-384.

39. Parker PA, Davis JW, Latini DM, et al. Relationship between illness uncertainty, anxiety, fear of progression and quality of life in men with favourable-risk prostate cancer undergoing active surveillance. *BJU Int.* 2016;117(3):469-477.

40. Pearce SM, Wang CH, Victorson DE, et al. A Longitudinal Study of Predictors of Sexual Dysfunction in Men on Active Surveillance for Prostate Cancer. *Sex Med.* 2015;3(3):156-164.

41. Pirl WF, Greer JA, Goode M, Smith MR. Prospective study of depression and fatigue in men with advanced prostate cancer receiving hormone therapy. *Psychooncology.* 2008;17(2):148-153.

42. Punnen S, Cowan JE, Dunn LB, Shumay DM, Carroll PR, Cooperberg MR. A longitudinal study of anxiety, depression and distress as predictors of sexual and urinary quality of life in men with prostate cancer. *BJU Int.* 2013;112(2):E67-75.

43. Rosenfeld B, Roth AJ, Gandhi S, Penson D. Differences in health-related quality of life of prostate cancer patients based on stage of cancer. *Psychooncology.* 2004;13(11):800-807.

44. Ruane-McAteer E, Porter S, O'Sullivan J, Dempster M, Prue G. Investigating the psychological impact of active surveillance or active treatment in newly diagnosed favorable-risk prostate cancer patients: A 9-month longitudinal study. *Psychooncology.* 2019;28(8):1743-1752.

45. Sciarra A, Gentilucci A, Salciccia S, et al. Psychological and functional effect of different primary treatments for prostate cancer: A comparative prospective analysis. *Urol Oncol.* 2018;36(7):340.e347-340.e321.

46. Shahinian VB, Kuo YF, Freeman JL, Goodwin JS. Risk of the "androgen deprivation syndrome" in men receiving androgen deprivation for prostate cancer. *Arch Intern Med.* 2006;166(4):465-471.

47. Sharpley CF, Bitsika V, Denham JW. Factors associated with feelings of loss of masculinity in men with prostate cancer in the RADAR trial. *Psycho-Oncology.* 2014;23(5):524-530.

48. Sharpley CF, Christie DR, Bitsika V, Miller BJ. Trajectories of total depression and depressive symptoms in prostate cancer patients receiving six months of hormone therapy. *Psychooncology.* 2017;26(1):60-66.

49. Shin D, Shim SR, Kim CH. Changes in Beck Depression Inventory scores in prostate cancer patients undergoing androgen deprivation therapy or prostatectomy. *PLoS One.* 2020;15(6):e0234264.

50. Steineck G, Helgesen F, Adolfsson J, et al. Quality of life after radical prostatectomy or watchful waiting. *N Engl J Med.* 2002;347(11):790-796.

51. Tan HJ, Marks LS, Hoyt MA, et al. The Relationship between Intolerance of Uncertainty and Anxiety in Men on Active Surveillance for Prostate Cancer. *J Urol.* 2016;195(6):1724-1730.

52. Tavlarides AM, Ames SC, Thiel DD, Diehl NN, Parker AS. Baseline and follow-up association of the MAX-PC in Men with newly diagnosed prostate cancer. *Psychooncology.* 2015;24(4):451-457.

53. Thornton AA, Perez MA, Oh S, Crocitto L. Optimism and prostate cancer-specific expectations predict better quality of life after robotic prostatectomy. *J Clin Psychol Med Settings.* 2012;19(2):165-176.

54. Timilshina N, Breunis H, Alibhai S. Impact of androgen deprivation therapy on depressive symptoms in men with nonmetastatic prostate cancer. *Cancer.* 2012;118(7):1940-1945.

55. Tully KH, Nguyen DD, Herzog P, et al. Risk of Dementia and Depression in Young and Middle-aged Men Presenting with Nonmetastatic Prostate Cancer Treated with Androgen Deprivation Therapy. *Eur Urol Oncol.* 2021;4(1):66-72.

56. van den Bergh RC, Korfage IJ, Roobol MJ, et al. Sexual function with localized prostate cancer: active surveillance vs radical therapy. *BJU Int.* 2012;110(7):1032-1039.

57. van den Driessche H, Mattelaer P, van Oyen P, et al. Changes in Body Image in Patients with Prostate Cancer over 2 Years of Treatment with a Gonadotropin-Releasing Hormone Analogue (Triptorelin): Results from a Belgian Non-Interventional Study. *Drugs Real World Outcomes.* 2016;3(2):183-190.

58. van Stam MA, Aaronson NK, Bosch J, et al. Patient-reported Outcomes Following Treatment of Localised Prostate Cancer and Their Association with Regret About Treatment Choices. *Eur Urol Oncol.* 2020;3(1):21-31.

59. van Tol-Geerdink JJ, Leer JW, van Lin EN, Schimmel EC, Stalmeier PF. Depression related to (neo)adjuvant hormonal therapy for prostate cancer. *Radiother Oncol.* 2011;98(2):203-206.

60. Venderbos LD, van den Bergh RC, Roobol MJ, et al. A longitudinal study on the impact of active surveillance for prostate cancer on anxiety and distress levels. *Psychooncology.* 2015;24(3):348-354.

61. Zhang Z, Yang L, Xie D, Shi H, Li G, Yu D. Depressive symptoms are found to be potential adverse effects of androgen deprivation therapy in older prostate cancer patients: A 15-month prospective, observational study. *Psychooncology.* 2017;26(12):2238-2244.

62. van den Bergh RC, Essink-Bot ML, Roobol MJ, Schröder FH, Bangma CH, Steyerberg EW. Do anxiety and distress increase during active surveillance for low risk prostate cancer? *J Urol.* 2010;183(5):1786-1791.
