# Supplementary material for: Prognostic factors for mental wellbeing in prostate cancer: A systematic review and meta‐analysis
Source: Psychooncology. 2023 Oct 3;32(11):1644–59. doi: 10.1002/pon.6225 (PMC10946963; doi:10.1002/pon.6225)
Supplement: Supplementary file 6 — Supporting Information S6 [file PON-32-1644-s004.docx]

**Supplementary Material 6: Individual Study Data for Prognostic Factors for Depression**

| **Study** | **Country** | **N. Patients** | **Mean Age** | **Treatment** | **Stage** | **Diagnostic Criteria** | **Prognostic Factor Results** | |
| --- | --- | --- | --- | --- | --- | --- | --- | --- |
| Boeri 2018 | Italy | 811 | Not stated | RP | Not stated | BDI | Patient Factors  *Univariate analysis*  Age – OR 1.87, CI (1.26-4.21), p 0.007  Marital status (yes vs no) – OR 0.61, CI (0.31-1.23), p 0.71  Oncological Factors  Depression scores over time – Increasing mean scores at 6 months (p <0.001), 12 months (p 0.002), 24 months (p 0.033) and 36 months (p <0.001)  Treatment Factors  Open vs robotic approach (univariate analysis) – OR 4.38, CI (2.42-7.93), p <0.89)  Open vs robotic approach (multivariate analysis) – OR 5.01, CI (2.35-10.64), p <0.001  Post-op ED (univariate analysis) – OR 2.77, CI (1.18-6.54), p 0.02  Post-op ED (multivariate analysis) – OR 7.31, CI (1.53-4.98), p 0.013  Post-op urinary continence (univariate analysis) – OR 0.75, CI (0.40-1.41), p 0.38  Post-op urinary continence (multivariate analysis) – OR 0.71, CI (0.32-1.59), p 0.417 | |
| Chen 2015 | Taiwan | 12872 | 74 | RP, RT, or ADT | Not stated | ICD-9 | *Univariate Analysis*  Patient Factors  Age 65< - HR 1.55, CI (0.85-1.55), p 0.364  Co-morbidities:  Diabetes Mellitus – HR 1.68, CI (1.09-1.68), p 0.007  Congestive Heart Failure – HR 2.02, CI (1.13-2.02), p 0.006  Chronic Kidney Disease – HR 1.59, CI (0.92-1.59), p 0.178  Liver disease – HR 1.41, CI (0.86-1.41), p 0.444  Rheumatological disease – HR 2.43, CI (1.01-2.43), p 0.047  Coronary artery disease – HR 2.52, CI (1.09-2.52), p 0.019  Cerebrovascular disease – HR 2.06, CI (1.34-2.06), p <0.001  Geographic region: Urban vs rural – HR 1.20, CI (0.76-1.20), p 0.693, Suburban vs rural – HR 1.19, CI (0.59-1.19), p 0.314  Income NTD (New Taiwan Dollar): 20,000 – 39,999 NTD/month vs <20,000 NTD/month – HR 1.14, CI (0.72-1.14), p 0.395, >40,000 NTD/month vs <20,000 NTD/month – HR 1.16, CI (0.69-1.16), p 0.412  Treatment Factors  ADT Type:  Orchiectomy – HR 1.21, CI (0.89-1.64), p 0.226  Adrenal blockers – HR 1.12, CI (0.83-1.51), p 0.459  Anti-androgens – HR 1.09, CI (0.87-1.37), p 0.458  Oestrogen – HR 1.03, CI (0.77-1.37), p 0.861  GnRH – HR 0.97, CI (0.75-1.24), p 0.780  RP – HR 0.94, CI (0.70-1.28), p 0.702  RT – HR 1.55, CI (1.04-2.31), p 0.032  *Multivariate Analysis – adjusted for age, co-morbidities, geographic regions, monthly income level*  Patient Factors  Co-morbidities:  Diabetes Mellitus – HR 1.26, CI (1.01-1.57), p 0.040  Congestive Heart Failure – HR 1.31, CI (0.97-1.77), p 0.073  Rheumatological disease – HR 1.43, CI (0.92-2.22), p 0.113  Coronary artery disease – HR 1.40, CI (0.91-2.15), p 0.124  Cerebrovascular disease – HR 1.55, CI (1.24-1.93), p <0.001  Treatment Factors  RT – HR 1.53, CI (1.03-2.29), p 0.036 | |
| Chen 2020 | Taiwan | 71 | 72.5 | ADT | Stage I-IV | PHQ-9 | Patient Factors  Age – p 0.15  Oncological Factors  Gleason score – p 0.13  Initial PSA – p 0.13 | |
| Chien 2018a | Taiwan | 117 | 66.7 | RP, RT + ADT, ADT or other | T1-4 | HADS | Patient Factors  Age – β -0.011 SE 0.035, p 0.750  Education (high school) – β 0.487 0.SE 0.642, p 0.569  Education (college) – β -0.383 SE 0.672, p 0.448  Religion (Taoism) – β 0.339 SE 0.762, p 0.657  Religion (Buddhism) – β -0.566 SE 0.637, p 0.374  Religion (other) – β 1.913 SE 1.019, p 0.061  Employment status – β -0.894 SE 0.532, p 0.093  Coping behaviours (problem) – β 0.008 SE 0.017, p 0.663  Coping behaviours (affective) – β 0.092 SE 0.018, p 0.001  Recent PSA – β 0.00003 SE 0.00002, p 0.047  Prostate symptoms (urinary) – β -0.049 SE 0.009, p 0.001  Prostate symptoms (bowel) – β -0.037 SE 0.010, p 0.001  Prostate symptoms (sexual) – β -0.036 SE 0.008, p 0.001  Prostate symptoms (hormonal) – β -0.117 SE 0.017, p 0.001  Appraisals of disease (benign) – β -0.322 SE 0.057, p 0.001  Appraisals of disease (challenge) – β -0.216 SE 0.067, p 0.001  Appraisals of disease (threat) – β 0.481 SE 0.026, p 0.001  Appraisals of disease (harm) – β 0.254 SE 0.057, p 001  Oncological Factors  Cancer stage (T3) – β 0.745 SE 0.584, p 0.202  Cancer stage (T4) – β -1.015 SE 0.784, p 0.195  Treatment Factors  RT and ADT vs RP – β 0.108 SE 0.709, p 0.879  ADT alone vs RP – β -0.657SE 0.707, p 0.353 | |
| Chung 2017 | Taiwan | 868 | 74.1 | ADT | Not stated | ICD-9 | *All HR are adjusted for all other variables*  Patient Factors  Geographic regions (ref: Northern): Central – HR 1.31, CI (0.60-2.88), p 0.501, Southern – HR 0.96, CI (0.43-2.17), p 0.924, Eastern – HR 2.73, CI (10.95), p 0.157  Monthly income NTD (New Taiwan Dollar): 0 NTD – 15,840 NTD (ref), 15,841 NTD – 25,000 NTD – HR 0.85, CI (0.41-1.77), p 0.665, 25,000 NTD < - HR 0.44, CI (0.10-1.90), p 0.274  Urbanisation Level (1 most urbanised, 5 least urbanised), (ref: 1): 2 – HR 0.59, CI (0.23-1.54), p 0.282, 3 – HR 1.14, CI (0.42-3.12), p 0.796, 4 – HR 2.16, CI (0.83-5.65), p 0.114, 5 – HR 1.71, CI (0.58-5.03), p 0.327  Co-morbidities:  Hypertension – HR 2.05, CI (0.92-4.57), p 0.081  Diabetes – HR 1.13, CI (0.58-2.20), p 0.717  Hyperlipidaemia – HR 0.89, CI (0.47-1.70), p 0.721  Coronary heart disease – HR 1.18, CI (0.64-2.18), p 0.605  Stroke – HR 2.09, CI (1.14-3.86), p 0.018  Treatment Factors  ADT – HR 1.93, CI (1.03-3.62), p 0.041 | |
| De Cerqueira 2015 | Brazil | 30 | 64.73 | FC, RT and AS | Gleason score <6 | BDI | Treatment Factors  Treatment type – p 0.4860 | |
| Deka 2019 | USA | 39,965 | 66.81 | RT or RT and ADT | T1-T3 | ICD-9 | Patient Factors  Age (<60 y vs 60-70 y) – HR 0.61, CI (0.51-0.71), p <0.001  Age (<60 y vs 70-80 y) – HR 0.49, CI (0.40-0.59), p <0.001  Age (<60 y vs 80< y) – HR 0.62, CI (0.39-0.97), p 0.03  Charlson Co-morbidity Index (0 vs 1) – HR 1.10, CI (0.94-1.30), p 0.22  Charlson Co-morbidity Index (0 vs 2<) - HR 1.21, CI (0.89-1.63), p 0.21  Race (White vs African American) – HR 1.00, CI (0.86-1.18), p 0.17  Race (White vs Other) – HR 0.92, CI (0.66-1.30), p 0.94  Region (West vs Midwest) – HR 0.85, CI (0.67-1.05), p 0.13  Region (West vs South) – HR 0.94, CI (0.77-1.15), p 0.58  Region (West vs Northeast) – HR 0.80, CI (0.67-0.96), p 0.01  Bachelor’s degree (ref: <10%)  10%-20% - HR 1.09, CI (0.93-1.29), p 0.27  20%-30% - HR 1.07, CI (0.85-1.34), p 0.56  >30% - HR 1.41, CI (0.99-2.00), p 0.05  Income (<$30,000 vs $30,000-$60,000) – HR 1.18, CI (0.91-1.51), p 0.19  Income (<$30,000 vs $60,000-$100,000) – HR 1.16, CI (0.86-1.57), p 0.31  Income (<$30,000 vs >$100,000) – HR 1.28, CI (0.76-2.13), p 0.34  Year of Diagnosis (ref: 2001)  2002 – HR 1.04, CI (0.74-1.45), p 0.81  2003 – HR 1.05, CI (0.75-1.46), p 0.77  2004 – HR 1.10, CI (0.79-1.53), p 0.56  2005 – HR 1.15, CI (0.83-1.60), p 0.37  2006 – HR 1.11, CI (0.80-1.54), p 0.53  2007 – HR 1.03, CI (0.74-1.44), p 0.84  2008 – HR 1.27, CI (0.91-1.77), p 0.14  2009 – HR 1.07, CI (0.76-1.52), p 0.67  2010 – HR 1.04, CI (0.73-1.48), p 0.83  2011 – HR 0.87, CI (0.59-1.28), p 0.49  2012 – HR 0.86, CI (0.56-1.31), p 0.48  2013 – HR 0.62, CI (0.37-1.04), p 0.07  2014 – HR 0.90, CI (0.55-1.46), p 0.67  2015 – HR 0.28, CI (0.08-0.92), p 0.03  SSRI – HR 1.56, CI (1.18-2.06), p 0.002  SNRI – HR 0.94, CI (0.69-1.31), p 0.73  Atypical antidepressant – HR 1.43, CI (0.99-2.06), p 0.05  Serotonin modulator – HR 1.66, CI (1.20-2.31), p 0.002  Tricyclic antidepressant – HR 1.17, CI (0.85-1.60), p 0.33  Alcohol abuse – HR 0.96, CI (0.62-1.48), p 0.86  Substance abuse – HR 1.55, CI (0.82-2.93), p 0.17  Cigarette use – HR 0.68, CI (0.36-1.31), p 0.25  Treatment Factors  ADT use (no ADT vs ADT) – HR 1.50, CI (1.32-1.71), p <0.01  *Sensitivity Analysis – adjusted for propensity score in FG model*  ADT use (No ADT vs ADT) – HR 1.51, CI (1.32-1.71), p <0.01  *Sensitivity Analysis – adjusted for time-varying ADT exposure*  ADT use (No ADT vs ADT) – HR 1.51, CI (1.32-1.72), p <0.01  *Sensitivity Analysis – excluded patients developing depression one year of their diagnosis (n = 39,929)*  ADT use (No ADT vs ADT) – HR 1.50, CI (1.31-1.71), p <0.01 | |
| Dinh 2016 | USA | 78552 | 75.7 | ADT | Stage I-III | ICD-9 | *All adjusted HR (adjustment for education level, income, population density, year. Of diagnosis, primary treatment with RP, primary treatment with RP*  Patient Factors  Age at diagnosis – HR 1.04, CI (1.03-1.04), p <0.001  Race (black vs white) – HR 0.74, CI (0.66-0.82), p <0.001  Race (other vs white) – HR 0.71, CI (0.61-0.82), p <0.001  Marital status (unmarried vs married) – HR 1.23, CI (1.15-1.32), p <0.001  Charlson Co-morbidity Index (1 vs 0) – HR 1.23, CI (1.15-1.32), p <0.001  Charlson Co-morbidity Index (2< vs 0) – HR 1.92, CI (1.77-2.09), p <0.001  Oncological Factors  Cancer stage (Stage I-II and Gleason 7-10 vs Stage I-II and Gleason 2-6) – HR 1.09, CI (1.02-1.17), p 0.009  Cancer stage (Stage III and Gleason of any grade vs Stage I-II and Gleason 2-6) – HR 1.08, CI (0.96-1.20), p 0.20  Treatment Factors  Months of ADT (None vs 1-6 months) - HR 1.12, CI (1.03-1.21), p 0.008  Months of ADT (None vs 7-11 months) – HR 1.26, CI (1.15-1.39), p <0.001  Months of ADT (None vs 12< months) – HR 1.37, CI (1.26-1.49), p <0.001 | |
| Donovan 2016 | UK | 1643 | 62 | AS, RP or RT | T1-2 | HADS | Treatment Factors  Treatment (AS/RP/RT) – p 0.85 | |
| Duarte 2002 | Portugal | 292 | 67.8 | AS, Curative (RP, RT), Palliative (ADT +/- Chemotherapy) | T1-T4, N0-N1, M0-M1 | HADS-D | *Adjustment for age and education*  *Baseline Characteristics*  Patient Factors  Age (<65 y vs >65 y) – OR 1.44, CI (0.29-7.06)  Education (1-4 y vs 5-9 y) – OR 0.37, CI (0.04-3.36)  Education (1-4 y vs 10-12 y) – OR 0.66, CI (0.08-5.72)  Education (1-4 y vs >12 y) – OR 0.55, CI (0.06-4.93)  Living alone (yes vs no) – OR 6.35, CI (1.43-28.30)  Employment (employed vs retired) – OR 1.06, CI (0.09-12.12)  Smoking (Never smoking vs Ex-smoker) – OR 0.57, CI (0.13-2.47)  Smoking (Never smoking vs Current smoker) – OR 1.29, CI (0.13-12.58)  Alcohol consumption (>20 vs 10-20 g/day) – OR 1.68, CI (0.36-7.70)  Vegetable consumption (=<5 portions/day vs >=5 portions/day) – OR 1.08, CI (0.26-4.48)  Physical activity (=<150 mins/week vs >=150 min/week) – OR 2.39, CI (0.58-9.89)  BMI (ref: 18.5-24.9 kg/m^2^)  25-29.9 kg/m^2^ – OR 0.58, CI (0.08-4.27)  >=30 kg/m^2^ – OR 2.11, CI (0.33-13.36)  Comorbidities (None vs 1-2) – OR 0.35, CI (0.07-1.66)  Comorbidities (None vs >=3) – OR 0.70, CI (0.11-4.46)  Oncological Factors  Metastases present (No vs yes) – OR 0.68, CI (0.08-5.67)  Treatment Factors  Treatment (AS vs Curative intent) – OR 0.39, CI (0.04-3.44)  Treatment (AS vs Palliative intent) - OR 0.49, CI (0.03-9.03)  *One year after diagnosis Characteristics*  Patient Factors  Age (<65 y vs >65 y) – OR 0.74, CI (0.28-1.92)  Education (1-4 y vs 5-9 y) – OR 0.22, CI (0.05-1.07)  Education (1-4 y vs 10-12 y) – OR 0.97, CI (0.29-3.23)  Living alone (no vs yes) – OR 1.54, CI (0.31-7.62)  Residence area (Urban vs rural) – OR 0.30, CI (0.04-2.39)  Employment (employed vs Sick leave/unemployed) – OR 0.46, CI (0.05-4.32)  Employment (employed vs Retired) – OR 0.38, CI (0.08-1.77)  Smoking (never smoking vs Ex-smoker) – OR 1.77, CI (0.62-5.07)  Smoking (never smoking vs Current smoker) – OR 1.55, CI (0.27-8.88)  Alcohol consumption (>20 vs 10-20 g/day) – OR 1.44, CI (0.50-4.15)  Vegetable consumption (=<5 portions/day vs >=5 portions/day) – OR 1.06, CI (0.38-2.96)  Physical activity (=<150 min/week vs >=150 min/week) – OR 0.58, CI (0.22-1.54)  BMI (ref: 18.5-24.9 kg/m^2^)  25-29.9 kg/m^2^ – OR 2.06, CI (0.40-10.64)  >=30 kg/m^2^ – OR 4.15, CI (0.73-23.57)  Comorbidities (none vs 1-2) – OR 0.48, CI (0.15-1.54)  Comorbidities (non vs >=3) – OR 1.53, CI (0.41-5.65)  Oncological Factors  Metastases present (no vs yes) – OR 1.19, CI (0.31-4.53)  Treatment Factors  Treatment (AS vs Curative intent) – OR 0.46, CI (0.09-2.36)  Treatment (As vs Palliative intent) - OR 0.52, CI (0.06-4.63) | |
| Egger 2018 | Australia | 341 | 69 | AS, RP or RT (EBRT or BT) | T1-T4 | HADS | Treatment Factors  AS vs RP – MD -0.9, NS  RT and HDR – MD 1.8, NS  LDR – MD 5.8, NS | |
| Ene 2006 | Sweden | 140 | 63.1 | RP | Stage I-III | HAD-D | Treatment Factors  Baseline vs 3 months post-surgery – Mean difference -0.4 p <0.05 | |
| Erim 2019a | USA | 1024 | Not stated | Not stated | T1-T3 | SF-12 | Patient Factors  Age – OR 0.98 (0.97-0.99), p <0.05  Race (White American vs African American) – OR 1.33, CI (1.07-1.66), p <0.05  Marital status (Current vs previously/never) – OR 1.19, CI (0.91-1.56), p 0.40  Education (High school or less vs High School or above) – OR 0.95, CI (0.75-1.21), p 0.14  Current Tobacco (No vs Yes) – OR 1.13, CI (0.85-1.50), p 0.14  Alcohol (No vs Yes) – OR 1.07, CI (0.87-1.33)  Residence (Urban vs Rural) – OR 0.88, CI (0.69-1.12)  Existing diagnosis of depression (No vs Yes) – OR 2.44, CI (1.82-3.27), p <0.01  Received emotional support (No vs Yes) – OR 1.11, CI (0.73-1.68)  Employment status (Retired vs employed) – OR 1.28, CI (1.00-1.64)  Employment status (Retried vs unemployed) – OR 1.74, CI (1.18-2.56), p <0.01  Income ($70K vs $40-70K) – OR 1.26, CI (0.96-1.65)  Income ($70K vs $20-40K) – OR 1.28, CI (0.93-1.76)  Income ($70K vs <$20K) – OR 1.57, CI (1.03-2.39), p <0.05  Charlson Co-morbidity index (0-1 vs 2+) – OR 1.59, CI (1.28-1.96), p 0.01  Adherent to exercise recommendations (No vs Yes) – OR 0.67, CI (1.55-0.82)  Oncological Factors  Time since PC diagnosis (Ref: 0-12 months)  Time since PC diagnosis (13-24 months) – OR 0.73, CI (0.43-1.23)  Time since PC diagnosis (25-36 months) – OR 0.57, CI (0.34-0.95), p <0.05  Time since PC diagnosis (37-48 months) – OR 0.44, CI (0.27-0.69), p <0.01  Time since PC diagnosis (49-60 months) – OR 0.42, CI (0.26-0.67)  Time since PC diagnosis (61-72 months) – OR 0.34, CI (0.20-0.58)  Time since PC diagnosis (73-84 months) – OR 0.33, CI (0.16-0.66)  Cancer stage (T1 a-c vs T2/T3 a-c) – OR 1.03, CI (0.84-1.26)  Treatment Factors  Treatment decisional regret (No vs Yes) – OR 3.31, CI (2.23-4.92), p <0.01 | |
| Erim 2016b | USA | 805 | Not stated | Not stated | T1-T3 | SF-12 | *At Diagnosis*  Patient Factors  Age – OR 0.95, CI (0.93-0.98), p <0.01  Race (European American vs African American) – OR 0.63, CI (0.42-0.94), p <0.05  Marital status (Currently vs previously/never) – OR 1.23, CI (0.78-1.91)  Education (High school or below vs High school or above) – OR 1.56, CI (1.03-2.35), p <0.05  Residence (Urban vs rural) – OR 1.24, CI (0.80.1.92)  Severity of depression (No vs plausible) – OR 2.1, CI (1.43-3.09), p <0.01  Severity of depression (No vs mild) – OR 5.08, CI (3.42-7.55), p <0.01  Severity of depression (No vs moderate/severe) – OR 13.04, CI (7.48-22.73), p <0.01  Employment status (Retired/unemployed vs employed) – OR 0.63, CI (0.43-0.93), p <0.05  Income ($70K vs $40-70K) – OR 0.8, CI (0.52-1.22)  Income ($70K vs $20-40K) – OR 0.84, CI (0.50-1.42)  Income ($70K vs <$20K) – OR 0.93, CI (0.50-1.71)  Charlson Co-morbidity index (0-1 vs 2+) – OR 1.08, CI (0.78-1.49)  Visits to primary care (<3 visits per year vs >3 visits per year) – OR 1.62, CI (1.24-2.12), p <0.01  Health insurance coverage (insured vs uninsured) – OR 0.57, CI (0.22-1.50)  Oncological Factors  Time since PC diagnosis (Ref: 13-24 months)  Time since PC diagnosis (25-36 months) – OR 0.61, CI (0.33-1.11),  Time since PC diagnosis (37-48 months) – OR 0.53, CI (0.28-0.98), p <0.05  Time since PC diagnosis (49-60 months) – OR 0.50, CI (0.26-0.98), p <0.05  Time since PC diagnosis (61-72 months) – OR 0.44, CI (0.22-0.88), p <0.05  Time since PC diagnosis (73-84 months) – OR 0.42, CI (0.18-1.01)  Cancer stage (T1 a-c vs T2/T3 a-c) – OR 0.82, CI (0.56-1.21)  *At Treatment*  Patient Factors  Age – OR 0.99, CI (0.95-1.04)  Race (European American vs African American) – OR 0.70, CI (0.35-1.42)  Marital status (Currently vs previously/never) – OR 1.12, CI (0.50-2.51)  Education (High school or below vs High school or above) – OR 1.14, CI (0.54-2.41)  Residence (Urban vs rural) – OR 1.42, CI (0.68-2.93)  Severity of depression (No vs plausible) – OR 2.19, CI (0.97-4.97)  Severity of depression (No vs mild) – OR 3.77, CI (1.72-8.22), p <0.01  Severity of depression (No vs moderate/severe) – OR 4.93, CI (2.22-10.96), p <0.01  Employment status (Retired/unemployed vs employed) – OR 0.49, CI (0.25-0.96), p <0.05  Income ($70K vs $40-70K) – OR 1.05, CI (0.44-2.50)  Income ($70K vs $20-40K) – OR 0.65, CI (0.44-2.50)  Income ($70K vs <$20K) – OR 0.46, CI (0.14-1.53)  Charlson Co-morbidity index (0-1 vs 2+) – OR 0.72, CI (0.42-1.25)  Visits to primary care (<3 visits per year vs >3 visits per year) – OR 1.42, CI (0.82-2.45)  Health insurance coverage (insured vs uninsured) – OR 2.07, CI (0.44-9.70)  Oncological Factors  Time since PC diagnosis (Ref: 13-24 months)  Time since PC diagnosis (25-36 months) – OR 0.14, CI (0.04-0.51), p <0.01  Time since PC diagnosis (37-48 months) – OR 0.22, CI (0.06-0.84), p <0.05  Time since PC diagnosis (49-60 months) – OR 0.22, CI (0.05-0.91), p <0.05  Time since PC diagnosis (61-72 months) – OR 0.17, CI (0.04-0.76), p <0.05  Time since PC diagnosis (73-84 months) – OR 0.27, CI (0.04-1.83)  Cancer stage (T1 a-c vs T2/T3 a-c) – OR 0.64, CI (0.32-1.26) | |
| Ferhava 2021 | Canada | 2445 | 68.1 | ADT | Not stated | PHQ-9 | *Univariate models*  Patient Factors  Age (per 10 year decrease) – OR 1.23, CI (1.04-1.42), p 0.016  Education (>secondary vs secondary) – OR 1.27, CI (0.91-1.77), p 0.17  Education (> secondary vs primary) – OR 1.89, CI (1.27-2.81), p 0.002  Tobacco (Never vs former) – OR 1.37, CI (0.99-1.90), p 0.061  Tobacco (Never vs current) – OR 3.17, CI (2.08-4.83), p <0.0001  Alcohol (Never vs former) – OR 3.05, CI (1.68-5.65), p <0.0001  Alcohol (Never vs current) – OR 1.26, CI (0.75-2.12), p 0.38  Employment – OR 1.06, CI (0.79-1.42), p 0.71  Poor performance status (ECOG >1) – OR 5.34, CI (3.86-7.37), p <0.0001  Pre-existing diagnosis of depression or anxiety – OR 3.67, CI (2.61-5.16), p <0.0001  Multiple morbidity – OR 1.35, CI (0.86-2.12), p 0.19  BMI (<30kg/m squared vs >30 kg/m squared) – OR 1.34, CI (0.99-1.82)), p 0.055  Erectile dysfunction (IIEF score <22) – OR 1.53, CI (0.91-2.57), p 0.11)  Multiple Morbidity – OR 1.75, CI (1.20-2.54), p 0.004  Oncological Factors  PC risk (Low/intermediate vs High) – OR 1.73, CI (1.29-2.33), p <0.0001  Gleason 3+4 or higher – OR 1.34, CI (1.00-1.79), p 0.054  Metastatic disease – OR 1.45, CI (0.89-2.36), p 0.14  *Multivariate models – erectile dysfunction, metastatic disease and Gleason score omitted from model*  Patient Factors  Age (per 10 year decrease) – OR 1.38, CI (1.16-1.60), p 0.001  Education (>secondary vs secondary) – OR 0.84, CI (0.56-1.26), p 0.40  Education (>secondary vs primary) – OR 1.42, CI (0.89-2.26), p 0.14  Tobacco (Never vs former) – OR 1.34, CI (0.91-1.96), p 0.14  Tobacco (Never vs current) – OR 2.77, CI (1.66-4.58), p <0.0001  Alcohol (Never vs former) – OR 2.63, CI (1.33-5.20), p 0.006  Alcohol (Never vs current) – OR 1.30, CI (0.72-2.34), p 0.38  Poor performance status (ECOG >1) – OR 5.01, CI (3.49-7.20), p <0.0001)  Pre-existing diagnosis of depression or anxiety – OR 3.64, CI (2.42-5.48), p <0.0001  BMI (<30 kg/m squared vs >30 kg/m squared) – OR 1.07, CI (0.75-1.51), p 0.72  Multiple morbidity – OR 1.35, CI (0.86-2.12), p 0.19  Oncological Factors  PC risk (Low/intermediate vs High) – OR 1.49, CI (1.05-2.12), p 0.027 | |
| Friberg 2021 | Denmark | 5570 | Not stated | RP | Not stated | ICD-10 | *All hazard ratios are adjusted for age-group at surgery, income quintile, cohabitation status, year group at surgery and co-morbidity index at diagnosis*  Patient Factors  Income quintile at diagnosis (ref: 5 highest)  1 (lowest) – HR 1.44, CI (1.13-1.84)  2 – HR 1.21, CI (0.96-1.53)  3 – HR 1.18, CI (0.95-1.46)  4 – HR 0.83, CI (0.67-1.04)  Cohabitation status (no vs yes) – HR 0.78, CI (0.65-0.95)  Year group at diagnosis (ref: 1998-2005):  2006-2007 – HR 0.87, CI (0.71-1.06)  2008-2009 – HR 0.76, CI (0.62-0.92)  2010-2011 – HR 0.61, CI (0.49-0.76)  Charlson Comorbidity index at diagnosis (0 vs 1) – HR 1.02, CI (0.81-1.29)  Charlson Comorbidity index at diagnosis (0 vs 2+) - HR 1.87, CI (1.25-2.79)  Treatment Factors  ADT (no ADT vs ADT) – HR 1.76, CI (1.35-2.30)  Salvage radiation (no RT vs RT) – HR 1.26, CI (0.98-1.61) | |
| Gagliano-Juca 2018 | USA | 37 | 67 | ADT | Not stated | PHQ-9 | Treatment Factors  Treatment (ADT/RP) – MD 0.93 (0.04-1.82), p 0.042 | |
| Hervouet 2013 | Canada | 60 | 70.04 | ADT + RT, RT only | Stage I-III | Structured Clinical Interview based on DSM-IV for Depressive Disorder | Oncological Factor  Time since diagnosis (0-16 Months) - p=0.35  Treatment Factors  ADT vs ADT + RT at 4 and 6 Months - p>0.1 | |
| Hoyt 2015 | USA | 66 | 65.76 | RP or RT | Gleason 6 | CES-D | Patient Factors  Age – SE 0.00, β -0.25, NS  Sexual activity – SE 0.09, β 0.16, NS  Sexual self-schema – SE 0.00, β -0.01, NS  Sexual functioning – SE 0.00, β -0.25, p<0.05 | |
| Hu 2021 | China | 194 | 62.5 | RP | T2-T4, N0-N1 | SDS | Treatment Factors  Time since discharge (36 months every 3 months) SDS score – P <0.001  Time since discharge (36 months every 3 months) depression rate – P <0.001 | |
| Kohler 2014 | Germany | 329 | 65.3 | RP | T1-T3 | HADS | Treatment Factors  RP post-surgery time 3 months vs 12 months – p <0.005 | |
| Luckenbaugh 2022 | USA | 2742 | 64 | RT, RP, ADT, AS | T1-T2 | CES-D | Treatment Factors  RP vs AS – MD -0.5, CI (-1.0-0.0), p 0.067  RT + ADT vs AS – MD -0.1, CI (-0.9-0.7), p 0.78  RT vs AS – MD 0, CI (-0.6-0.6), p 0.959 | |
| Lee 2015 | USA | 122 | 67 | ADT or RP | Not stated | CES-D | Treatment Factors  ADT or RP at baseline – p <0.05  ADT or RP at 6 months – p<0.01 | |
| Lev 2009 | USA | 159 | 55.8 | RT + ADT, RT + Seeds or RP | Gleason 5-10 | CES-D | Oncological Factors  Depression scores from baseline vs. 6 and 12 months – NS across all treatment groups  Treatment Factors  RP vs. Brachy - NS  RP vs. RT - NS  RT vs Brachy - NS | |
| Mohamed 2012 | USA | 869 | 65.45 | RT/Brachytherapy, RP | T1-T2, N0, M0 | CES-D | Treatment Factors  Type of Treatment RP vs RT – β 0.20 p=0.001 | |
| Nordin 2001 | Sweden | 99 | Not stated | Not stated | T4, N1, M1 | HADS | Treatment Factors  Baseline vs 6 months - NS  Non-advanced PC baseline vs 6 months – NS  Advanced PC baseline vs 6 months – p 0.05 | |
| Pirl 2008 | USA | 52 | 62 | ADT | Not stated | BDI | Treatment Factors  ADT at 6 months – SE 0.73, p 0.23 and ADT at 12 months – SE 0.67, p 0.49 | |
| Punnen 2013 | USA | 679 | 60.1 | AS or RP | Not stated | PHQ-9 | Treatment Factors  Baseline RP vs AS – p 0.65  1-3 years RP vs AS – p 0.77 | |
| Rosenfeld 2004 | USA | 341 | 71.2 | RP, Brachytherapy, RT, or ADT | Localised (T1-2), locally advanced (T3-4) and metastatic | HADS | Oncological Factors  Cancer staging (localised/locally advanced/metastatic disease) – p 0.34 | |
| Ruane McAteer 2019 | UK | 54 | 62.75 | AS or Active Treatment | Gleason 6-7 | CES-D | Treatment Factors  AS vs Active Treatment – p 0.114 | |
| Sciarra 2018 | Italy | 220 | 65.3 | AS, RP or RT | T1-T3, N0, M0 | HADS and PHQ-9 | *HADS-D – Multivariate analysis*  Patient Factors  Age – β -0.0062 CI (-0.05-0.04), p 0.789  Education – β 0.5199 CI (0.11-0.93), p 0.014  Family status – β -0.5609 CI (0.10-0.12), p 0.014  Employment β1.0466 – CI (0.283-1.81), p 0.008  Smoker – β 0.1975 CI (-0.46-0.86), p 0.558  BMI – β 0.0848 CI (0.01-0.16), p 0.037  IPSS – β 0.0211 CI (-0.05-0.10), p 0.587  ILEF-5 – β0.0526 CI (0.003-0.10), p 0.587  Oncological Factors  PSA – β 0.0026 CI (-0.03-0.04), p 0.884  Gleason score β -0.1601 – CI (-0.59-0.27), p 0.469  Treatment Factors  AS – β 2.6053CI (-1.78-6.98), p 0.246  RP – β 2.5628 CI (-1.41-6.53), p 0.208  RT – β 1.495 CI (-2.78-5.77), p 0.494 | *PHQ-9 – Multivariate analysis*  Patient Factors  Age – β -0.0072 CI (-0.03-0.02), p 0.608  Education – β -0.0097 CI (-0.26-0.24), p 0.940  Family status – β -0.0486 CI (-0.32-0.22), p 0.725  Employment – β 0.8289 CI (0.36-1.29), p 0.001  Smoker – β 0.0172 CI (-0.38-0.42), p 0.933  BMI – β 0.0495 CI (0.002-0.10), p 0.045  IPSS – β -0.0247 CI (-0.07-0.02), p 0.297  ILEF-5 – β 0.0374 CI (0.007-0.07), p 0.056  Oncological Factors  PSA – β -0.0118 CI (-0.03-0.01), p 0.278  Gleason score – β -0.2314 CI (-0.49-0.03), p 0.087  Treatment Factors  AS – β 0.2004 CI (-2.47-2.87), p 0.883  RP – β -0.2542 CI (-2.68-2.17), p 0.837  RT – β -0.5846 CI (-3.19-2.02), p 0.660 |
| Shahinian 2006 | USA | 50613 | 75 | ADT | Stage I-IV | ICD-9 | *Adjustment for comorbidities, tumour characteristics and age*  Treatment Factors  ADT use (ADT vs no ADT) – RR 1.08, CI (1.02-1.15) (adjusted), 1.37, CI (1.30-1.44) (unadjusted) | |
| Sharpley 2016 | Australia | 102 | 71.78 | HT | T1-T4, N0-N1, M0 | SDS | Treatment Factors  Time of HT treatment SDS score:   - Overall time (9 months) – p<0.001 - 3 months vs 6 months – p<0.001   3 months vs 9 months – p<0.001 | |
| Shin 2020 | Korea | 107 | Not stated | ADT or RP | Not stated | Beck’s Depression Inventory | Patient Factors  Age - p 0.227  BMI – p 0.021  Testosterone – p 0.034  IIEF score – p 0.897  Oncological Factors  PSA – p 0.643  Gleason score (<7) – p 0.410  Gleason score (8+) – p 0.146 | |
| Steineck 2002 | Sweden | 326 | 64.4 | RP or WW | T1-T2 | CES-D | Treatment Factors  RP vs WW – RR 0.6, CI (0.3-1.3) | |
| Timilshina 2012 | Canada | 257 | 69.1 | ADT | T1-T3 | GDS | Treatment Factors  ADT use at 3 months – p 0.42  ADT use at 6 months – p 0.25  ADT use at 12 months – p 0.19 | |
| Tully 2021 | USA | 325 | Not stated | ADT | Not stated | ICD-9 | *Adjustment for patient demographics and comorbidities*  Treatment Factors   - ADT use (ADT vs no ADT) – HR 2.07, CI (1.34-3.19), p <0.001 | |
| Van Den Bergh 2010 | Netherlands | 129 | 64.6 | AS | Non-palpable or localised | CES-D | Treatment Factors  Time since diagnosis (9 months) – p 0.929 | |
| Van den Bergh 2012 | Netherlands | 266 | 65.04 | AS, RP or RT | T1-T2 | CES-D | Treatment Factors  AS vs RP – p 0.138  AS vs RT – p 0.017  AS vs combined – p 0.026 | |
| Van Tol-Geerdink 2011 | Netherlands | 288 | 69 | ADT | T1-T3, N0, M0 | HADS | Treatment Factors  Neo-adjuvant ADT – p<0.01 | |
| Venderbos 2015 | Netherlands | 150 | 64.6 | AS | Non-palpable or localised | CES-D | Treatment Factors  Time of AS – p 0.65 | |
| Zhang 2017 | China | 146 | 70.4 | ADT, RP | T1-T3 | SDS | Patient Factors  Smoking – OR 13.661, CI (1.722-108.386), p 0.013  Alcohol – OR 6.868, CI (1.038-45.445), p 0.046  Exercise – OR 0.209, CI (0.028-1.562), p 0.127  Living with Children – OR 0.105, CI (0.011-0.959), p 0.046 | |
| *Index: ADT Androgen Deprivation Therapy, AS Active Surveillance, BDI Beck’s Depression Inventory, BMI Body Mass Index, CES-D Centre of Epidemiologic Studies Depression Score, DSM-IV Diagnostic and Statistical Manual of Mental Disorders IV, CI confidence Interval, FC Focal Cryoablation, GDS Geriatric Depression Scale, HADS Hospital Anxiety Depression Score, HAD-D Hospital Anxiety Depression – Depression, ICD-9 International Classification of Disease 9, ICD-10 International Classification of Disease 10, IIEF-5 International Index of Erectile Dysfunction-5, IPSS International Prostate Symptom Score, MD Mean Difference, NS Not Significant, OR Odds Ratio, PC Prostate Cancer, PHQ-9 Patient Health Questionnaire-9, PSA Prostate Specific Antigen, RP Radical Prostatectomy RR Risk Ratio, RT Radiotherapy, SDS Zung Self rating Depression Scale, SE Standard Error, , SF-12 Short Form 12, TNM Cancer Staging (Tumour, Node, Metastasis), WW Watchful Waiting.* | | | | | | | | |
