# Supplementary material for: Prognostic factors for mental wellbeing in prostate cancer: A systematic review and meta‐analysis
Source: Psychooncology. 2023 Oct 3;32(11):1644–59. doi: 10.1002/pon.6225 (PMC10946963; doi:10.1002/pon.6225)
Supplement: Supplementary file 7 — Supporting Information S7 [file PON-32-1644-s005.docx]

**Supplementary Material 7: Individual Study Data for Prognostic Factors for Anxiety**

| **Study** | **Country** | **N. Patients** | **Mean Age** | **Treatment** | **Stage** | **Diagnostic Criteria** | **Prognostic Factor Results** |
| --- | --- | --- | --- | --- | --- | --- | --- |
| Alvisi 2020 | Italy | 236 | 64.4 | AS | T1-T2 | MAX-PC | Patient Factors  Extraversion – OR 1.9  Anxious preoccupation – OR 4.4  Prostate Symptoms – OR 0.46 |
| Chien 2018a | Taiwan | 117 | 66.7 | RP, RT + ADT, ADT, other | T1-T4 | HADS | Patient Factors  Age – β -0.063 SE 0.033, p 0.054  Education (High school) – β 0.818 SE 0.610, p 0.179  Education (college) – β 0.430 SE 0.631, p 0.496  Religion (Taoism) – β 0.999 SE 0.703, p 0.156  Religion (Buddhism) – β -0.227 SE 0.632, p 0.720  Religion (other) – β 2.365 SE 1.157, p 0.041  Employment status – β -0.160 SE 0.496, p 0.747  Coping behaviours (problem) – β 0.030 SE 0.015, p 0.052  Coping behaviours (affective) – β 0.117 SE 0.013, p 0.001  Prostate symptoms (urinary) – β -.053 SE 0.011, p 0.001  Prostate symptoms (bowel) – β -0.046 SE 0.011, p 0.001  Prostate symptoms (sexual) – β -0.025 SE 0.007, p 0.001  Prostate symptoms (hormonal) – β -0.112 SE 0.013, p 0.001  Appraisals of disease (benign) – β -0.372 SE 0.044, p 0.001  Appraisals of disease (challenge) – β -0.176 SE 0.054, p 001  Appraisals of disease (threat) – β 0.521 SE 0.038, p 0.001  Appraisals of disease (harm) – β 0.245 SE 0.024, p 0.001  Oncological Factors  Cancer stage (T3) – β 0.915 SE 0.572, p 0.110  Cancer stage (T4) – β -1.173 SE 0.689, p 0.088  Recent PSA – β 0.00008 SE 0.00002, p 0.001  Treatment Factors  RT + ADT vs RP – β -0.199-SE 0.680, p 0.70  ADT alone vs RP– β -1.403 SE 0.652, p 0.031 |
| Chien 2018b | Taiwan | 48 | 67 | RP or RT | Stage I-III | Memorial Anxiety Scale | Patient Factors  Age – β -0.03, SE <0.001, p<0.001  Religion (ref: no) - β -0.225, SE 0.203, NS  Employment status (ref: unemployed) – β -0.171, SE 0.170, NS  Education (ref: below primary school) – junior/senior high school (beta 0.452, SE 0.240, NS), college and above (β 0.289, SE 0.238, NS)  Self perceived health status – β -0.004, SE 0.004, NS  Relationship satisfaction – β -0.242, SE 0.096, p<0.05  Physical symptoms – urinary (β -0.001, SE 0.002), bowel (β -0.008, SE 0.003, p<0.01), sexual (β -0.001, SE 0.003, NS), hormonal (β -0.012, SE 0.004, p<0.01)  Oncological Factors  Cancer TNM stage T3 (ref: T2) – β 0.153, SE 0.173, NS  Treatment Factors  Radiotherapy vs RP (ref) – β 0.153, SE 0.173, NS |
| De Cerqueira 2015 | Brazil | 30 | 64.73 | FC, RT and AS | Gleason score <6 | BAI | Treatment Factors  Treatment type – p 0.6780 |
| Dinh 2017 | USA | 78552 | 75.7 | ADT | Stage I-III | ICD-9 | *HR – adjusted for features including education level, income, population density, year of diagnosis, primary treatment with RT/RP*  Patient Factors  Age – HR 1.01, CI (1.00-1.02), p 0.015  Ethnicity (Black vs white) – HR 0.65, CI (0.56-0.75), p 0.001  Marriage status (unmarried vs married) – HR 1.22, CI (1.12-1.34), p <0.001  Married status (unknown vs married) – HR 1.21, CI (1.07-1.37), p 0.003  Charlson comorbidity index (0 vs 1) – HR 1.17, CI (1.07-1.28), p 0.001  Charlson comorbidity index (score 0 vs 2+) – HR 1.51, CI (1.35-1.69), p 0.001  Oncological Factors  Cancer Stage (Gleason 7-10 vs Gleason 2-6) – HR 1.02, CI (0.94-1.11), p 0.699  Cancer stage (Stage III any Gleason score vs Stage I-II Gleason score 2-6) - HR 0.87, CI (0.96-1.20), p 0.064  Treatment Factors  Months of ADT (1-6) – HR 1.05, CI (0.95-1.15), p 0.380  Months of ADT (7-11) – HR 1.06, CI (0.93-1.20), p 0.388  Months of ADT (12+) – HR 1.16, CI (1.04-1.29), p 0.010 |
| Donovan 2016 | UK | 1643 | 62 | AS, RP and RT | T1-T2 | HADS | Treatment Factors  AS/RP/RT – p 0.20 |
| Dordoni 2022 | Italy | 823 | 64 | RP, RT or AS | T1-T2 | MAX-PC | Patient Factors  Country (Italy vs The Netherlands) – LR 0.92, CI (-0.38-2.23), p 0.16  Education (Ref: primary or secondary education) (overall p 0.5):  Professional school or College – LR -0.53, CI (-1.41-0.34)  University and post-degree – LR -0.42, CI (-1.44-0.59)  Relationship Status (Not married vs married/living together) – LR 0.3, CI (-0.86-1.46), p 0.6  Treatment Factors  Years on AS – LR 0.2, CI (-0.36-0.76), p 0.5  Years on AS (Italian men vs Dutch men) – LR -0.95, CI (-1.72-0.17), p 0.017 |
| Duarte 2022 | Portugal | 292 | 67.8 | AS, Curative (RP, RT), Palliative (ADT +/- Chemotherapy) | T1-T4, N0-N1, M0-M1 | HAD-A | *Adjustment for age and education*  *Baseline Characteristics*  Patient Factors  Age (<65 y vs >65 y) – OR 1.51, CI (0.54-4.20)  Education (1-4 y vs 5-9 y) – OR 0.60, CI (0.16-2.29)  Education (1-4 y vs 10-12 y) – OR 1.04, CI (0.28-3.95)  Education (1-4 y vs >12 y) – OR 1.57, CI (0.50-4.92)  Living alone (no vs yes) – OR 1.03, CI (0.22-4.77)  Residence area (urban vs rural) – OR 2.80, CI (0.91-8.58)  Employment (employed vs Sick leave/unemployed) – OR 1.73, CI (0.17-17.65)  Employment (employed vs Retired) – OR 1.69, CI (0.32-8.86)  Smoking (never smoking vs Ex-smoker) – OR 0.49, CI (0.19-1.23)  Smoking (never smoking vs Current smoker) – OR 0.43, CI (0.05-3.58)  Alcohol consumption (>20 vs 10-20 g/day) – OR 0.90, CI (0.36-2.25)  Vegetable consumption (=<5 portions/day vs >=5 portions/day) – OR 1.11, CI (0.45-2.76)  Physical activity (=<150 min/week vs >=150 min/week) – OR 0.90, CI (0.38-2.16)  BMI (ref: 18.5-24.9 kg/m^2^)  25-29.9 kg/m^2^ – OR 0.33, CI (0.12-0.91)  >=30 kg/m^2^ – OR 0.34, CI (0.09-1.34)  Comorbidities (none vs 1-2) – OR 0.57, CI (0.22-1.51)  Comorbidities (none vs >=3) – OR 0.13, CI (0.02-1.14)  Oncological Factors  Metastases present – OR 0.51, CI (0.11-2.31)  Treatment Factors  Treatment (AS vs Curative intent) – OR 0.51, CI (0.11-2.31)  Treatment (AS vs Palliative intent) - OR 1.32, CI (0.16-10.64)  *One year after diagnosis Characteristics*  Patient Factors  Age (<65 y vs >65 y) – OR 0.85, CI (0.35-2.06)  Education (1-4 y vs 5-9 y) – OR 0.42, CI (0.11-1.62)  Education (1-4 y vs 10-12 y) – OR 1.82, CI (0.63-5.25)  Education (1-4 y vs >12 y) – OR 0.41, CI (0.08-1.95)  Living alone (no vs yes) – OR 1.03, CI (0.22-4.86)  Residence area (urban vs rural) – OR 1.81, CI (0.54-6.05)  Employment (employed vs Sick leave/unemployed) – OR 1.48, CI (0.26-8.56)  Employment (employed vs Retired) – OR 0.67, CI (0.16-2.87)  Smoking (never smoking vs Ex-smoker) – OR 1.02, CI (0.41-2.52)  Smoking (never smoking vs Current smoker) – OR 0.43, CI (0.05-3.66)  Alcohol consumption (>20 vs 10-20 g/day) – OR 1.02, CI (0.39-2.66)  Vegetable consumption (=<5 portion/day vs >=5 portions/day) – OR 0.94, CI (0.37-2.42)  Physical activity (<=150 min/week vs >=150 min/week) – OR 0.46, CI (0.18-1.16)  BMI (ref: 18.5-24.9 kg/m^2^)  25-29.9 kg/m^2^ – OR 1.59, CI (0.48-5.21)  >=30 kg/m^2^ – OR 0.99, CI (0.21-4.67)  Comorbidities (none vs 1-2) – OR 0.48, CI (0.19-1.25)  Comorbidities (none vs >=3) – OR 0.26, CI (0.05-1.32)  Oncological Factors  Metastases present – OR 2.07, CI (0.69-6.22)  Treatment Factors  Treatment (AS vs Curative intent) – OR 0.62, CI (0.13-3.11)  Treatment (AS vs Palliative intent) - OR 1.10, CI (0.15-8.09) |
| Egger 2018 | Australia | 341 | 69 | AS, RP or RT (EBRT or BT) | T1-T4 | HADS | Treatment Factors  AS vs RP – MD -0.8, NS  RT and HDR – MD 4.9, NS  LDR – MD 5.5, NS |
| Ene 2006 | Sweden | 140 | 63.1 | RP | Stage I-III | HAD-A | Treatment Factors  Baseline vs 3 months post-surgery – Mean difference -2.0 p <0.001 |
| Fleshner 2012 | Canada | 302 | 65.1 | AS or ADT | T1-T2 | MAX-PC | Oncological Factors  Time since initiation of treatment ADT baseline vs. 3 years - Mean difference -1.5 SE 0.65, p 0.036  Treatment Factors  Dutasteride vs. Control – NS |
| Gagliano-Juca 2018 | USA | 37 | 67 | ADT | Not stated | STAI state and trait score | Treatment Factors  ADT/RP – MD 2.34, (-0.09-4.76), p 0.37 |
| Hu 2021 | China | 194 | 62.5 | RP | T2-T4, N0-N1 | SAS | Oncological Factors  Time since discharge (36 months every 3 months) SAS score – p<0.001  Time since discharge (36 months every 3 months) Anxiety rate – p =0.004 |
| Kohler 2014 | Germany | 329 | 65.3 | RP | T1-T3 | HADS | Treatment Factors  RP post-surgery time 3, 6 and 12 months – p <0.001 |
| Krupski 2005 | USA | 208 | 58.98 | RP, RT, or ADT | Not stated | 5-point Likert scale | Patient Factors  Ethnicity – p 0.08 |
| Marzouk 2018 | USA | 463 | 61 | AS | D’Amico low/intermediate risk prostate cancer | Memorial Anxiety Scale | Patient Factors  Age – OR 0.98, CI (0.96-1.01), p 0.3  Overall Health Score – OR 0.83, CI (0.74-0.93), p 0.002  Relationship status (single vs not single) – OR 0.69, CI (0.42-1.13), p 0.14  Visit Type (Non-biopsy vs Biopsy) – OR 1.26, CI (0.83-1.92), p 0.3  Oncological Factors  Number of positive core biopsies – OR 1.00, CI (0.83-1.19), p 1.00  Visit type (biopsy vs non-biopsy) – OR 1.26, CI (0.83-1.92), p 0.3  Gleason score (6 vs 7-8) – OR 0.43, CI (0.12-1.49), p 0.2  Family History of PC (No vs yes) – OR 1.03, CI (0.66-1.61), p 0.9 |
| Naha 2021 | USA | 302 | 65 | AS | Not stated | MAX-PC | Patient Factors  Age – p 0.01  Oncological Factors  PSA – p 0.11 |
| Nordin 2001 | Sweden | 99 | Not stated | Not stated | T4, N1, M1 | HADS | Treatment Factors  Baseline vs 6 months – mean difference -0.6, p=0.05  Non-advanced PC baseline vs 6 months – NS  Advanced PC baseline vs 6 months – NS |
| Pearce 2015 | USA | 195 | 66.5 | AS | <T2a, gleason <6 | MAX-PC | Treatment Factors  Time of AS (24 months) – p<0.0001 |
| Punnen 2013 | USA | 679 | 60.1 | AS or RP | Not stated | GAD-7 | Treatment Factors  Baseline RP vs AS – p 0.94  1 year RP vs AS – p 0.89  1-3 years RP vs AS – p 0.17 |
| Rosenfeld 2004 | USA | 341 | 71.2 | RP, Brachytherapy, RT, ADT, Chemotherapy, AS | Localised (T1-T2), Locally advanced (T3-T4), metastatic (T3-T4, N1-N3 or Ma-Mc) | HADS | Oncological Factors  Cancer stage (Localised disease, locally advanced, metastatic) – p 0.31 |
| Ruane McAteer 2019 | UK | 54 | 62.75 | AS or Active Treatment | Gleason 6-7 | MAX-PC and STAI-6 | Treatment Factors  AS vs Active Treatment MAX-PC – p 0.054  AS vs Active Treatment STAI-6 – p 0.614 |
| Sciarra 2018 | Italy | 220 | 65.3 | AS, RP or RT | T1-T3, N0, M0 | HADS | Patient Factors  Age – β -0.0062 CI (-0.02-0.09), p 0.215  Education – β 0.5199 CI (-0.40-0.62), p 0.679  Family status – β -0.5609 CI (-0.67-0.44), p 0.682  Employment – β 1.0466 CI (-1.14-0.77), p 0.707  Smoker – β 0.1975 CI (-1.62-0.03), p 0.060  BMI – β 0.0848 CI (-0.03-0.17), p 0.190  IPSS – β 0.0211 CI (-0.04-0.15), p 0.236  ILEF-5 – β 0.0526 CI (-0.05-0.07), p 0.678  Oncological Factors  PSA – β0.0026 CI (0.02-0.11), p 0.004  Gleason score – β -01601 CI (-0.09-0.99), p 0.104  Treatment Factors  AS – β 2.6053 CI (-6.57-4.41), p 0.701  RP – β 2.5628 CI (-5.60-4.36), p 0.806  RT – β 1.495 CI (-5.42-5.29), p 0.982 |
| Steineck 2002 | Sweden | 326 | 64.4 | RP or WW | T1-T2 | STAI-A | Treatment Factors  RP vs WW – RR 0.9, CI (0.5-1.8) |
| Tan 2016 | USA | 119 | 62.8 | AS | Not stated | HADS-A | Patient Factors  Age – p 0.636  Ethnicity – p 0.999  Married – p 0.517  Education – p 0.765  BMI – p 0.760  Co-morbidities – p 0.255  Positive family history – p 0.059  Existing depression – p 0.148  IPSS score – p 0.003  Greater intolerance of uncertainty – OR 1.15, CI (1.07-1.23)  *Longitudinal Analysis (multivariate analysis – adjustment for age, gender, race/ethnicity, education, marital status, comorbidity, FH of PC, depression and urinary symptoms and time since study entry):*  Age – OR 0.95, CI (0.87-1.02)  Race (non-white vs white) – OR 0.77, CI (0.14-4.06)  Education (college graduate vs non-college graduate) – OR 0.85, CI (0.16-4.49)  Marital status (married vs non-married) – OR 1.15, CI (0.29-4.59)  Co-morbidities (present vs none) – OR 2.14, CI (0.62-7.41)  Family History of PC – OR 0.95, CI (0.27-3.43)  Existing Depression – OR 2.48, CI (0.33-18.73)  Urinary symptoms (moderate/severe vs mild) – OR 3.44, CI (1.13-10.50)  IUS score – OR 1.15, CI (1.07-1.23)  Time since study entry – OR 0.95, CI (0.89-1.01)  Oncological Factors  Baseline PSA – p 0.701  Biopsy – p 0.600  Treatment Factors  Time of AS – OR 0.92, CI (0.85-1.01)  *Sensitivity Analysis for relationship between tolerance of uncertainty and anxiety – Longitudinal analysis*  Patient Factors  Index Models – OR 1.15, CI (1.07-1.23)  Index Models + men with intermediate risk disease and adjusting for PSA/Gleason score – OR 1.15, CI (1.07-1.24)  Index Models + interaction for FH of PC and IUS score – OR 1.20, CI (1.10-1.30)  Index Models + interaction for urinary symptoms and IUS Score – OR 1.07, CI (0.39-1.25)  Index Models + interaction for depression and IUS Score – OR 1.17, CI (1.09-1.25) |
| Tavlaride 2015 | USA | 350 | 63.8 | RP | T1-T3 | MAX-PC | Patient Factors  Age at treatment – p 0.094  Marital status – p 0.024  Race – p 0.054  History of erectile dysfunction – p 0.82  Diabetes – p 0.71  Positive family history – p 0.46  Oncological Factors  Pre-op PSA – p 0.1  PSA 6-18 months post-surgery – p -0.002  Gleason score – p 0.025  Cancer stage – p 0.004  Treatment Factors  Type of RP (retropubic or robotic) – p 1.00  Nerve sparing – p 0.95 |
| Van Den Bergh 2010 | Netherlands | 129 | 64.6 | AS | Non-palpable or localised | MAX-PC | Oncological Factors  Time since diagnosis (9 months) – p 0.550 |
| Van den Bergh 2012 | Netherlands | 266 | 65.0 | AS, RP and RT | T1-T2 | STAI-6 | Treatment Factors  AS vs RP – p 0.095  AS vs RT – p 0.261  AS vs combined – p 0.090 |
| Van Stam 2020 | Netherlands | 434 | 66.4 | AS, ADT and RT | T1-T2 | MAX-PC | Treatment Factors  Treatment Type – p 0.534  Time since treatment anxiety declined – p <0.001  AS continued vs discontinued – p >0.04 (NS)  RT with ADT vs RT without ADT – p >0.04 (NS) |
| Venderbos 2015 | Netherlands | 150 | 64.6 | AS | Non-palpable or localised | MAX-PC | Treatment Factors  Time of AS – p 0.33 |
| *Index: ADT Androgen Deprivation Therapy, AS Active Surveillance, BAI Beck’s Anxiety Inventory, BMI Body Mass Index, Score, CI confidence Interval, FC Focal Cryoablation, GAD-7 Generalised Anxiety Disorder assessment 7, HADS Hospital Anxiety Depression Score, HAD-A Hospital Anxiety Depression – Anxiety, ICD-9 International Classification of Disease 9, IIEF-5 International Index of Erectile Dysfunction-5, IPSS International Prostate Symptom Score, LR Likelihood Ratio, MAX-PC Memorial Anxiety Scale – Prostate Cancer, MD Mean Difference, NS Not Significant, OR Odds Ratio, PC Prostate Cancer, PSA Prostate Specific Antigen, RP Radical Prostatectomy RR Risk Ratio, RT Radiotherapy, SAS Zung Self reporting Anxiety Scale, SE Standard Error, SMD Standard Mean Difference, TNM Cancer Staging (Tumour, Node, Metastasis), WW Watchful Waiting.* | | | | | | | |
