# Supplementary material for: Prognostic factors for mental wellbeing in prostate cancer: A systematic review and meta‐analysis
Source: Psychooncology. 2023 Oct 3;32(11):1644–59. doi: 10.1002/pon.6225 (PMC10946963; doi:10.1002/pon.6225)
Supplement: Supplementary file 8 — Supporting Information S8 [file PON-32-1644-s007.docx]

**Supplementary Material 8 Individual Study Data for Prognostic Factors for Masculinity**

| **Study** | **Country** | **N. Patients** | **Mean Age** | **Treatment** | **Stage** | **Diagnostic Criteria** | **Prognostic Factor Results** |
| --- | --- | --- | --- | --- | --- | --- | --- |
| Dowrick 2018 | Australia | 540 | 62.2 | RP | T1-T3 | The Clark health worry and regret scale (Masculinity scale) | Patient Factors  Partner vs unpartnered – p 0.24 |
| Sharpley 2014 | Australia | 1070 | 67.5 | ADT | T2-T4 | EORTIC QLQ-PR25 | *At 18 months*  Patient Factors  Depression - SE 5.486, p 0.0001  Feeling tense – SE 3.321, p 0.001  Difficulty remembering things – SE 3.217, p 0.001  Tiredness – SE 2.831, p 0.005  *At 36 months*  Patient Factors  Depression – SE 3.372, p 0.001  Difficulty remembering things – SE 2.482, p 0.013  Tiredness – SE 5.385, p 0.000 |
| Thornton 2012 | USA | 83 | 61.83 | RP | T1-T3 | Prostate Cancer Related QofL scales (Masculine self-esteem 8 item scale) | Patient Factors  Optimism – beta 0.22, p <0.06, r2 0.13 |
| *Index: ADT Androgen Deprivation Therapy, EORTIC QLQ-PR25 European Organisation for Research and Treatment of Cancer Quality of Life Questionnaire, RP Radical Prostatectomy, SE Standard Error, TNM Cancer Staging (Tumour, Node, Metastasis)* | | | | | | | |
