# Supplementary material for: Prognostic factors for mental wellbeing in prostate cancer: A systematic review and meta‐analysis
Source: Psychooncology. 2023 Oct 3;32(11):1644–59. doi: 10.1002/pon.6225 (PMC10946963; doi:10.1002/pon.6225)
Supplement: Supplementary file 9 — Supporting Information S9 [file PON-32-1644-s006.docx]

**Supplementary Material 9: Individual Study Data for Prognostic Factors for Body Image**

| **Study** | **Country** | **N. Patients** | **Mean Age** | **Treatment** | **Stage** | **Diagnostic Criteria** | **Prognostic Factor Results** |
| --- | --- | --- | --- | --- | --- | --- | --- |
| Van den Driessche 2016 | Belgium | 145 | 77.8 | ADT | Gleason 6-10 | The body image scale | Treatment Factors  Months of ADT – 6M (p 0.018), 12M (p 0.008), 18M (p 0.001), 24M (p 0.068) |
| *Index: ADT Androgen Deprivation Therapy* | | | | | | | |
