# Supplementary material for: Prognostic factors for mental wellbeing in prostate cancer: A systematic review and meta‐analysis
Source: Psychooncology. 2023 Oct 3;32(11):1644–59. doi: 10.1002/pon.6225 (PMC10946963; doi:10.1002/pon.6225)
Supplement: Supplementary file 10 — Supporting Information S10 [file PON-32-1644-s008.docx]

**Supplementary Material 10: Individual Study Data for Prognostic Factors for Fear of Cancer Recurrence**

| **Study** | **Country** | **N. Patients** | **Mean Age** | **Treatment** | **Stage** | **Diagnostic Criteria** | **Prognostic Factor Results** |
| --- | --- | --- | --- | --- | --- | --- | --- |
| Alvisi 2020 | Italy | 236 | 64.4 | AS | T1-T2 | MAX-PC | Patient Factors  Neuroticism – OR 7.05  Functional wellbeing – OR 0.73 |
| Chien 2018b | Taiwan | 48 | 67 | RP or RT | Stage I-III | MAX-PC | Patient Factors  Age – beta -0.009, SE 0.007, NS  Religion (no vs yes) - beta -0.067, SE 0.1, NS  Employment status (unemployed vs employed) – beta -0.183, SE 0.095, NS  Education (below primary school vs junior/senior high school) - beta 0.042, SE 0.138, NS), college and above (beta 0.080, SE 0.139, NS)  Self perceived health status – beta -0.007, SE 0.003, p<0.05  Relationship satisfaction – beta -0.163, SE 0.085, NS  Physical symptoms – urinary (-0.004, SE 0.002, p<0.05), bowel (-0.002, SE 0.005, NS), sexual (beta <0.001, SE 0.002, NS), hormonal (beta -0.013, SE 0.005, p<0.05)  Oncological Factors  Cancer TNM stage (T2 vs T3) – beta 0.166, SE 0.092, NS  Treatment Factors  Radiotherapy vs RP – beta -0.175, SE 0.097, NS |
| Dordoni 2022 | Italy | 823 | 64 | RP, RT or AS | T1-T2 | MAX-PC (Fear of recurrence subscale) | Patient Factors  Country (Italy vs The Netherlands) – LR 3.38, CI (3.01-3.74), p <0.001  Education (Ref: primary or secondary education) (overall p 0.3):  Professional school or College – LR -0.20, CI (-0.46-0.06)  University and post-degree – LR -0.15, CI (-0.45-0.15)  Relationship Status (Not married vs married/living together) – LR -0.11, CI (-0.46-0.27), p 0.4  Treatment Factors  Years on AS – LR 0.03, CI (-0.21-0.27), p 0.8  Years on AS (Italian men vs Dutch men) – LR -0.66, CI (-1.03-0.30), p <0.001 |
| Egger 2018 | Australia | 341 | 69 | AS, RP or RT (EBRT or BT) | T1-T4 | Kornblith 5-item Fear of Cancer Progression Scale | Treatment Factors  AS vs RP – MD 2.7, NS  RT and HDR – MD -0.4, NS  LDR – MD 3.8, NS |
| Fleshner 2012 | Canada | 302 | 65.1 | AS or ADT | T1-T2 | MAX-PC | Treatment Factors  Dutasteride score change from baseline vs 3 years vs control – Mead difference -0.6 SE 0.19, p 0.017 |
| Hong 2010 | USA | 584 | Not stated | RP | T0-T4 | Komblith Fear of Recurrence survey | Patient Factors  Age – p 0.93  Race – p 0.16  Education – p 0.39  Relationship status – p 0.66  BMI – p 0.65  Oncological Factors  Time since diagnosis – reduced on repeated measure analysis p=0.019  PSA at diagnosis – p 0.63  Gleason score – p 0.12  Pathological T stage – p 0.68  Treatment Factors  Use of adjuvant therapy post RP – OR, 2.78 CI, 1.21–6.39 p=0.01  Positive Surgical margin – OR, 1.94 CI, 1.22–3.07 p <0.01 |
| Mehta 2003 | USA | 519 | 64.8 | RP or RT (EBRT or BT) | T1-T4, N1-N2, M1 | SF-36 | Patient Factors  *Multivariate regression*  General Health perception domain of SF-36 – p <0.001, r2 0.27  Mental health domain on SF-36 – p <0.001, r2 0.04  Oncological Factors  Gleason score, clinical stage, and treatment type – NS, total r2 0.015 |
| Meissner 2021 | Germany | 2417 | 69.5 | RP | Not stated | Fear of Progression Questionnaire (Fop-Q-SF) | *Multivariate analysis*  Patient Factors  Education (low vs intermediate) – OR 0.65, CI (0.37-1.12), p <0.001  Education (low vs high) – OR 0.56, CI (0.31-1.01), p 0.001  Education (low vs tertiary) – OR 0.23, CI (0.12-0.43), p 0.001  Age – OR 1.02, CI (0.99-1.06), p 0.228  Depression (No vs yes) – OR 1.45, CI (1.19-1.78), p 0.001  Anxiety (No vs yes) – OR 1.69, CI (1.37-2.09), p 0.001  FCR at baseline (No vs Yes) – OR 10.75, CI (6.18-18.72), p <0.001  Oncological Factors  Secondary cancer (No vs Yes) – OR 1.11, CI (0.59-2.10), p 0.758  Family History of PC (No vs Yes) – OR 2.24, CI (0.77-6.50), p 0.140  Treatment Factors  Years since RP – OR 1.10, CI (1.03-1.18), p 0.006 |
| Parker 2017 | USA | 180 | 67.2 | AS | Low risk (Gleason score 3+3) | 4 Item fear of cancer recurrence subscale of Memorial Anxiety Scale for Prostate Cancer | Patient Factors  Anxiety – p 0.0001  Race – p 0.222  Age – p 0.057  Testosterone levels – p 0.927  BMI – 0.461  Oncological Factors  Cancer stage – p 0.327  Gleason score – p 0.550  Family history of PC – p 0.162  Number of biopsies – p 0.751  PSA level – p 0.871 |
| Pearce 2017 | USA | 195 | 66.5 | AS | <T2a, Gleason <6 | MAX-PC (FCR subscale) | Treatment Factors:  Time of AS (24 months) – p = 0.6169 |
| *Index: ADT Androgen Deprivation Therapy, AS Active Surveillance, BMI Body Mass Index, BT Brachytherapy, CI Confidence Interval, EBRT External Beam Radiotherapy, MAX-PC Memorial Anxiety scale – Prostate Cancer, NS Non-Significant, OR Odds Ratio, PSA Prostate Specific Antigen, PC Prostate Cancer, RP Radical Prostatectomy, RT Radiotherapy, SE Standard Error, SF-36 Short Form 36 TNM Cancer Staging (Tumour, Node, Metastasis).* | | | | | | | |
