# Supplementary material for: Prognostic factors for mental wellbeing in prostate cancer: A systematic review and meta‐analysis
Source: Psychooncology. 2023 Oct 3;32(11):1644–59. doi: 10.1002/pon.6225 (PMC10946963; doi:10.1002/pon.6225)
Supplement: Supplementary file 11 — Supporting Information S11 [file PON-32-1644-s001.docx]

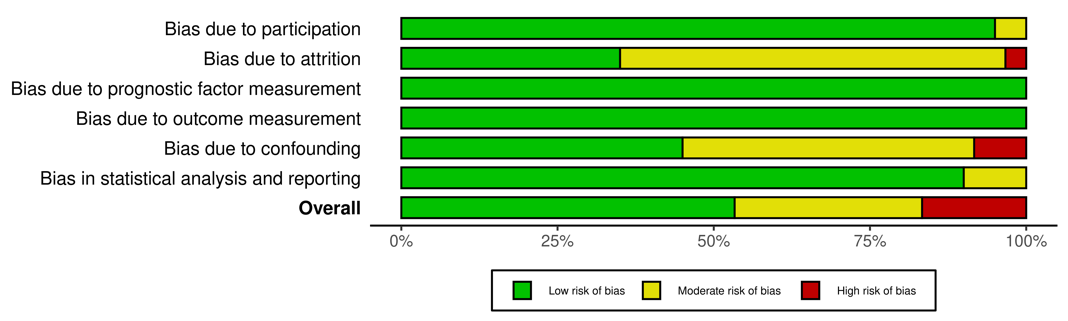

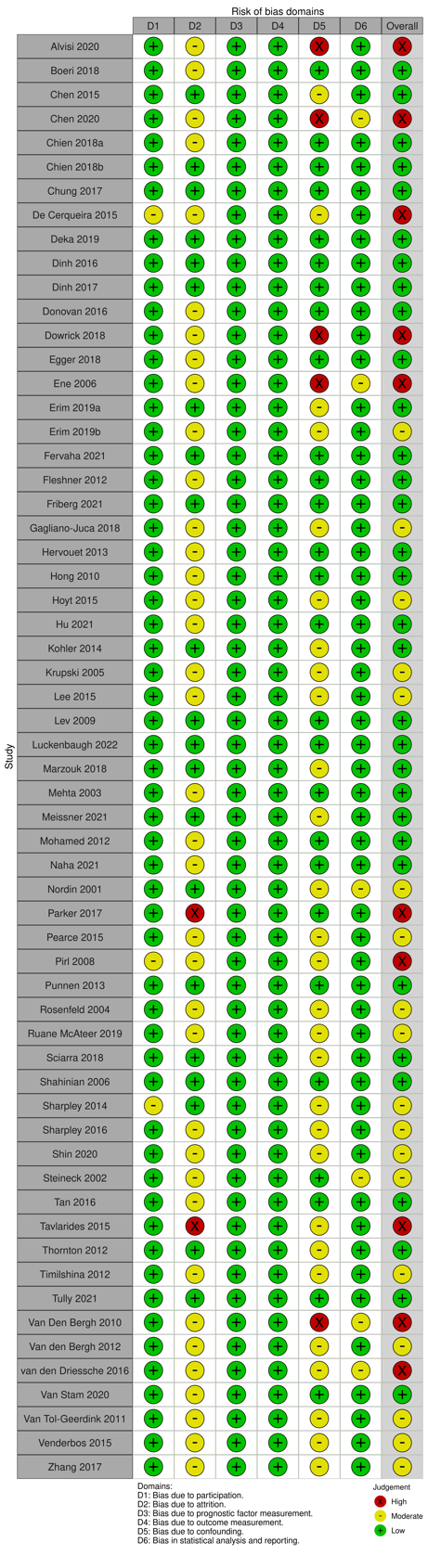


**Supplementary Material 11: A) Overall Risk of Bias (RoB) Graph. B) Individual Study Risk of Bias Table. *Categorization for RoB criteria: Green (low risk) - all domains were classified as having low RoB, or up to one moderate RoB. Red (high risk) - if one or more domains were classified as having high RoB, or ≥ 3 moderate RoB. All papers in between were classified as having moderate RoB (yellow).***
